# Stop codon readthrough alters the activity of a POU/Oct transcription factor during *Drosophila* development

**DOI:** 10.1186/s12915-021-01106-0

**Published:** 2021-09-03

**Authors:** Yunpo Zhao, Bo Gustav Lindberg, Shiva Seyedoleslami Esfahani, Xiongzhuo Tang, Stefano Piazza, Ylva Engström

**Affiliations:** 1 Department of Molecular Biosciences, The Wenner-Gren Institute, Stockholm University, SE-106 91 Stockholm, Sweden; 2 Present address: Department of Molecular Biology, Umeå University, SE-901 87 Umeå, SE Sweden; 3 Present address: Yale Stem Cell Center, Yale University School of Medicine, New Haven, Connecticut 06520 USA; 4 Present address: Research and Innovation Centre, Fondazione Edmund Mach, via E Mach 1, 38010 San Michele a/Adige, Italy

**Keywords:** Drosophila, Ecdysone, Gene expression, Intrinsically disordered region, Oct, POU, Metamorphosis, Steroidogenesis, Stop codon readthrough, Transcription factor

## Abstract

**Background:**

A number of cellular processes have evolved in metazoans that increase the proteome repertoire in relation to the genome, such as alternative splicing and translation recoding. Another such process, translational stop codon readthrough (SCR), generates C-terminally extended protein isoforms in many eukaryotes, including yeast, plants, insects, and humans. While comparative genome analyses have predicted the existence of programmed SCR in many species including humans, experimental proof of its functional consequences are scarce.

**Results:**

We show that SCR of the Drosophila POU/Oct transcription factor Ventral veins lacking/Drifter (Vvl/Dfr) mRNA is prevalent in certain tissues in vivo, reaching a rate of 50% in the larval prothoracic gland. Phylogenetically, the C-terminal extension is conserved and harbors intrinsically disordered regions and amino acid stretches implied in transcriptional activation. Elimination of Vvl/Dfr translational readthrough by CRISPR/Cas9 mutagenesis changed the expression of a large number of downstream genes involved in processes such as chromatin regulation, neurogenesis, development, and immune response. As a proof-of-principle, we demonstrate that the C-terminal extension of Vvl/Dfr is necessary for correct timing of pupariation, by increasing the capacity to regulate its target genes. The extended Vvl/Dfr isoform acts in synergy with the transcription factor Molting defective (Mld) to increase the expression and biosynthesis of the steroid hormone ecdysone, thereby advancing pupariation. Consequently, late-stage larval development was prolonged and metamorphosis delayed in vvl/dfr readthrough mutants.

**Conclusions:**

We demonstrate that translational recoding of a POU/Oct transcription factor takes place in a highly tissue-specific and temporally controlled manner. This dynamic and regulated recoding is necessary for normal expression of a large number of genes involved in many cellular and developmental processes. Loss of Vvl/Dfr translational readthrough negatively affects steroid hormone biosynthesis and delays larval development and progression into metamorphosis. Thus, this study demonstrates how SCR of a transcription factor can act as a developmental switch in a spatiotemporal manner, feeding into the timing of developmental transitions between different life-cycle stages.

**Graphical abstract:**

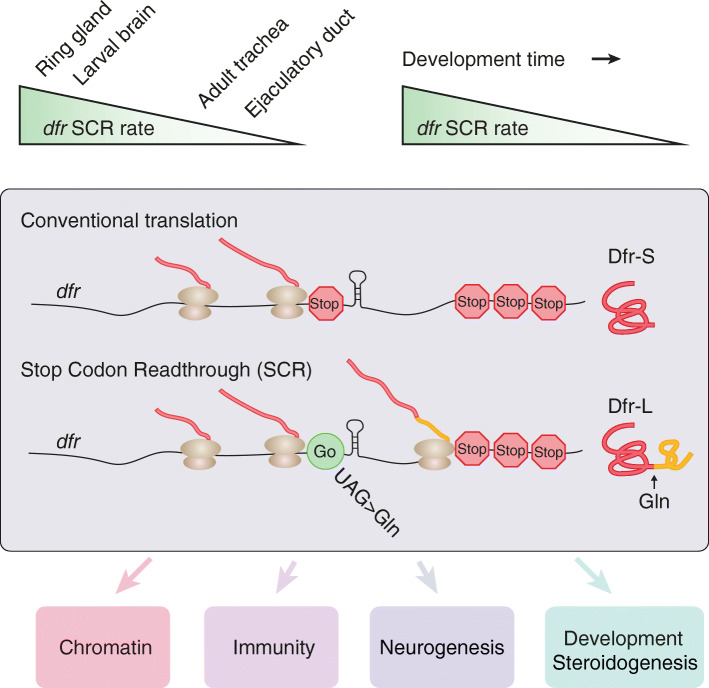

**Supplementary Information:**

The online version contains supplementary material available at 10.1186/s12915-021-01106-0.

## Background

Over the last decades of genome and metagenome sequencing projects, it has become apparent that the genetic code is non-universal, as a repertoire of alternative genetic decoding exists [1, 2]. Translation of mRNA by the ribosome continues until a stop codon (UAA, UAG, or UGA) is reached, which allows release factors, such as eukaryotic release factor 1 (eRF1), to recognize the stop codon and mediate termination [3]. Normally, the error rate of termination is less than 0.1%. If the interaction between eRF1 and mRNA is not efficient enough, near-cognate tRNAs (nc-tRNAs) are able to decode the stop codons as sense codons, resulting in stop codon readthrough (SCR). Initially characterized as an evolved common strategy of viruses to increase the protein repertoire without expanding the genome [4–6], SCR has recently been documented to occur in yeast, fungi, plants, insects, nematodes, and mammals [7]. Consequentially, protein isoforms with extended C-termini are generated. Added domains can provide signals for protein sorting, localization, stabilization/destabilization, and other functional domains [1].

The identity of the stop codon contributes to the relative termination fidelity, with UGA having the highest SCR potential, followed by UAG and UAA [8]. The base immediately 3′ of the stop codon also affects the readthrough, e.g., the level of UGA-C readthrough is higher than that of UGA-N [9]. In addition, RNA stem loop structures are enriched in the vicinity of potentially leaky stop codons and are both sufficient and necessary for readthrough of the *headcase (hdc)* gene in *Drosophila* [10]. In a few cases, RNA-binding proteins and miRNAs have been found to control the rate of SCR. For example, heterogeneous nuclear ribonucleoprotein (hnRNP) A2/B1 was shown to bind to a *cis*-acting element in *VEGF-A* 3′ untranslated region (UTR) and promote SCR [11]. Translational readthrough of the mammalian *AGO1* gene, encoding the Argonaute 1 (Ago1) protein, was recently found to be positively regulated by the let-7a miRNA upon binding 3′ of the canonical stop codon [12].

Several whole genome approaches have been used to identify genes undergoing SCR, such as ribosome profiling, phylogenetic analyses, and in silico identification of genes with specific stop codon contexts that are more prone to SCR [13]. For example, 57 human genes were identified with a favorable stop codon context and six of these were experimentally verified [14]. A recent annotation of SCR in nine vertebrate model organisms resulted in 13 genes exhibiting phylogenetically conserved C-terminal extensions, in total resulting in 94 SCR isoforms [15]. The most pervasive whole genome analyses of SCR have been carried out in insects, taking advantage of the complete genome sequences of numerous *Drosophila* and *Anopheles* species. Comparative genome analysis of 12 *Drosophila* species initially predicted that 149 genes undergo SCR [16]. In follow-up studies of 20 *Drosophila* and 21 *Anopheles* species, SCR was predicted for a total of 333 *Drosophila* and 353 *Anopheles* genes [17, 18]. Deep sequencing of ribosome-protected mRNA fragments (a.k.a. ribosome profiling) have provided genome-wide experimental validation of SCR in *Drosophila* [19] and in mammalian cells [7].

The functional importance of SCR has only been sparsely investigated. Early experimental studies identified the *Drosophila* genes for *Synapsin*, *kelch*, and *hdc* to produce alternative protein products through SCR [20–22]. SCR of *hdc* mRNA was shown to contribute to the regulation of tracheal development, providing one of the first evidences of an essential role of C-terminally extended proteins in *Drosophila* [10]. Recent studies in mammals have demonstrated that the functional and physiological significance of SCR is widespread in nature [23–25]. However, a clear understanding of the biological context and functional roles in vivo of SCR is nevertheless missing.

A comparative study, including 537 SCR candidate genes in *D. melanogaster,* showed that these gene products had a slight preference for nuclear localization and for involvement in biological processes related to regulation [26]. We noted that about 10% of the total number of transcription factors in *D. melanogaster* (75/703) have been predicted to undergo SCR [16–19]. Several of these transcription factors are involved in nervous system development or function. An interesting gene in this respect is *Drosophila ventral veins lacking (vvl)/ drifter (dfr)* (from hereon referred to as *dfr*), which is predicted to encode an unusually long C-terminal extension upon readthrough [17, 18]. Dfr is a member of the POU/Oct domain transcription factor family, including well-known regulators of embryonic and neural development, stem cell pluripotency, immunity, and cancer. Dfr plays profound roles during all stages of *Drosophila* development, such as regulation of embryonic brain and nervous system development, tracheogenesis, and adult epithelial immunity [27–30]. Its mammalian orthologs, POU3F1-POU3F4 regulate embryogenesis, neurogenesis, and neuronal differentiation and are referred to as the POU-class III of neural transcription factors [31–33]. POU/Oct proteins also control developmental transitions, such as POU1F1/Pit1, which in mammals regulates expression of several genes involved in pituitary development, expression of growth hormone and prolactin, and progression of puberty [34]. Similarly, it has been shown that *dfr* controls metamorphosis in insects by controlling the synthesis and release of steroid hormones from the prothoracic gland (PG) [35, 36], an endocrine organ with analogous functions to the mammalian pituitary gland.

In the present study, we show that the expression of a large number of genes are affected by the elimination of *dfr* SCR, pointing to an important role of the C-terminally extended form of the Dfr transcription factor, for processes such as development, metabolism, and immunity. Importantly, translational recoding of *dfr* is evolutionarily conserved and is regulated in a spatiotemportal manner, signifying its functional relevance. At the mechanistic level, we show that the kinetic profile of ecdysteroid biosynthesis, which acts as a timekeeper and coordinator of insect metamorphosis, requires SCR of *dfr*, thus demonstrating a critical role of SCR in timing developmental transitions. The evolutionary conservation of SCR in metazoans implies that it may serve as a general regulatory mechanism, playing more profound roles in cellular and organismal processes than previously anticipated.

## Results

### Translational stop codon readthrough of *dfr* mRNA

**Fig. 1:**
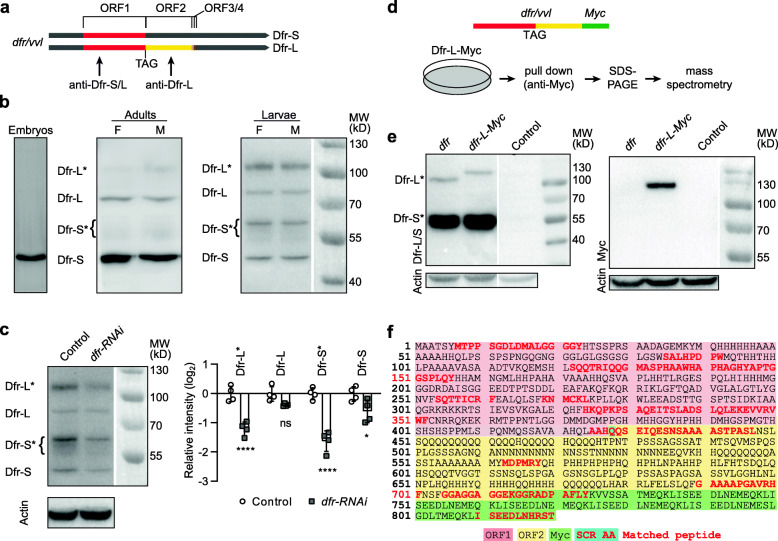
Translational readthrough of *dfr* mRNA produces two alternative Dfr isoforms. **a** Schematic representation of the intronless *dfr/vvl* gene. The *dfr* open reading frame 1 (ORF1, 427 amino acids, red), ending at the first UAG stop codon, is followed directly by a second frame (ORF2, 286 amino acids, yellow), flanked by 5′ and 3′ untranslated regions (gray). Translational readthrough of the UAG produces Dfr-L containing a C-terminal extension. The following two stop codons have also been predicted to undergo stop codon readthrough extending into ORFs 3 and 4 [17]. Two independent Dfr antibodies were used, one recognizing the common part of Dfr-S and Dfr-L (ORF1; anti-Dfr-S/L) [29] and another (anti-Dfr-L), specific for the C-terminal extension (ORF2). **b** Immunoblots on embryo, adult, and larval extracts, using anti-Dfr-S/L. **c** Temporal RNAi-mediated knockdown of *dfr* in larvae for 3 days using *Act-Gal4[ts]* crossed to *UAS-dfr-RNAi*, compared to control (driver only). Bars represent means+SE (*n* = 4). **d** Schematic illustration of expression and isolation of Dfr-L followed by mass spectrometry. An in-frame Dfr-L-Myc fusion construct was transfected into S2 cells and the SCR product was isolated by pull-down using anti-Myc, followed by separation by electrophoresis. Digestion was performed with chymotrypsin prior to analysis by nano liquid chromatography-mass spectrometry (nLC-MS/MS). **e** Immunoblots using protein extracts from S2 cells, untreated or transfected with *dfr* plasmids, with or without a C-terminal Myc-tag. Left panel, anti-Dfr-S/L; right panel, anti-Myc. Note that S2 cells do not express Dfr endogenously. **f** nLC-MS/MS and MASCOT analysis returned 15 peptides matching Dfr, of which 4 in ORF2. The stop codon was interpreted as a glutamine codon (highlighted in blue; see Additional file 2 for more details). MASCOT analysis against the Dfr-L-Myc fusion protein additionally returned peptides matching the Myc peptide (green) and the Dfr-L-Myc fusion

**Fig. 2:**
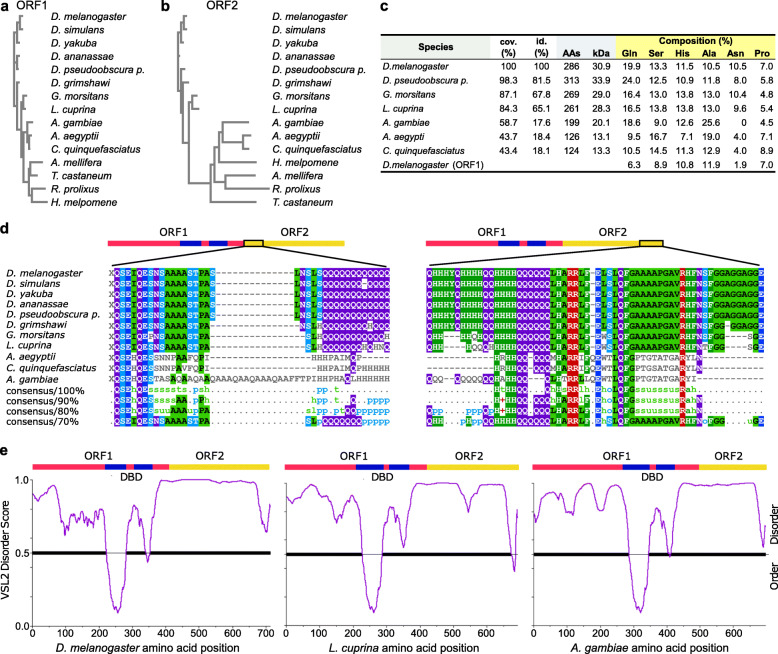
Phylogenetic analysis of Dfr ORF1/2. **a, b** Phylogenetic trees of Dfr ORF1 (**a**) and ORF2 (**b**), constructed using the neighbor-joining method, depicting real branch distances between ORFs of denoted species. Note that non-dipteran species included in the analysis has a comparatively short ORF2 and no predicted readthrough. **c** Comparison of amino acid sequences resulting from plausible translation of the ORF2. Percentage of selected amino acids in ORF2 of selected dipterans. The composition did only differ in *A. gambiae* (*p* = 0.046) when compared to *D. melanogaster*. cov., sequence coverage; id. amino acid identity; AAs, number of amino acids in ORF2; kDa, size of ORF2. **d** MView Sequence alignment of ORF2 in selected dipterans. Left panel, start of ORF2; right panel, C-terminal end of ORF2. **e** Disorder analysis of *Drosophila melanogaster* Dfr-L. The intrinsic disorder of Dfr-L was calculated by the VSL2 algorithm (http://www.pondr.com/). Schematic representation of ORF1 (red), ORF2 (yellow), and the DNA-binding domains (DBD, blue) are shown above the disorder graph. The horizontal bold line indicates 0.5 disorder score, above which the amino acid sequence is considered disordered

**Fig. 3:**
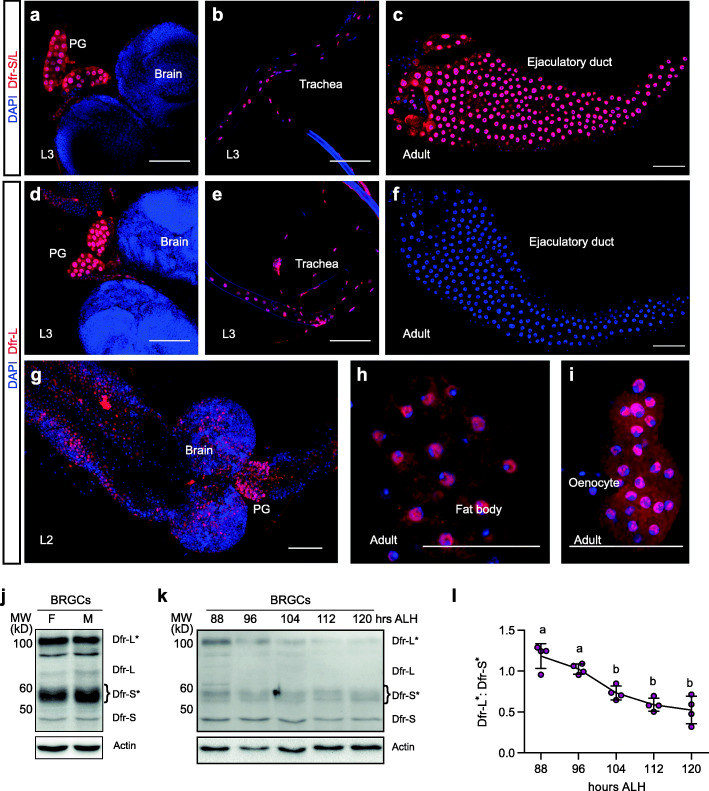
The relative frequency of *dfr* stop codon readthrough varies between tissues and development stages. **a–i** Confocal images of immunostaining, using the Dfr-S/L antibody recognizing both forms of Dfr (**a**–**c**), or the Dfr-L antibody specific for the C-terminal extension (**d**–**i**) of larval BRGCs (**a**, **c**, and **g**), larval trachea (**b** and **e**), adult male ejaculatory duct (**c** and **f**), adult fat body cells (**h**), and oenocytes (**i**). Scale bars represent 50 μm. **j–l** Immunoblot experiments using the Dfr-S/L antibody. Total protein was extracted from BRGCs of female and male wandering L3 larvae (**j**) or BRGCs of synchronized L3 larvae (**k**), at indicated time points (hours) ALH. For comparison, 88 h ALH is approximately equal to 40 h ALE in Fig. 6e. **j, k** Representative blots. Actin was used as loading control. **l** Quantification of the Dfr-S*:Dfr-L* ratio from (**k**). The relative concentration of Dfr-L* decreased from early L3 to late L3. The underlying line plot represent means; error bars depict SD (*n* = 4). Distinct letters above the points represent statistically significant difference (*p* < 0.05)

Phylogenetic analyses of codon substitution frequencies (PhyloCSF) and in silico identification of genes with specific stop codon contexts have pointed out *dfr* as a strong candidate for SCR [16, 17]. Similar to its orthologs, including human POU3F1-4, the *dfr* locus is intronless and has an unusually long 3′ UTR (2.4 kb; Fig. 1a). The first open reading frame (ORF) produces a 45.9-kDa protein (from hereon referred to as Dfr-S, with S depicting the short form). Predictions of *dfr* SCR into ORF2 [16, 17] would extend it by 286 amino acids to 76.8 kDa (referred to as Dfr-L, with L indicating the long form; Fig. 1a). The next two downstream stop codons have also been predicted to be subject to SCR, producing 78.1 and 79.9 kDa isoforms respectively (Fig. 1a) [17], but these were not experimentally verified in this study. We hypothesized that such a long, evolutionarily conserved, C-terminal extension would provide additional, or altogether different, properties to the protein (Fig. 2). The extent of SCR was analyzed using two different antibodies; one directed against the common ORF1, recognizing both Dfr-S and Dfr-L isoforms (anti-Dfr-S/L), and another directed against ORF2, specific for Dfr-L (anti-Dfr-L) (Fig. 1a). The latter antibody recognizes native Dfr-L in immunostaining experiments (Fig. 3), but does not bind the denatured protein. Therefore, the anti-Dfr-S/L antibody was used for the following immunoblot experiments. In embryos of mixed stages, we and others [27] only detected the Dfr-S isoform, indicating that *dfr* is not subject to prominent SCR during embryogenesis (Fig. 1b). In larval and adult extracts, however, Dfr-S/L incubation produced bands corresponding to the predicted molecular weights of both Dfr-S and Dfr-L. In addition, two bands of approximately 55–60 kDa and 105 kDa were consistently observed, from hereon referred to as Dfr-S* and Dfr-L*. To test whether each of the four bands was truly Dfr, we performed ubiquitous, temporal knockdown of *dfr* using temperature-sensitive Tubulin-Gal4 to drive UAS-*dfr*-RNAi (Fig. 1A) in adults. This resulted in a significantly decreased immunoblot intensity of Dfr-S/S* and Dfr-L* (Fig. 1c). Dfr-L was slightly, but not significantly altered, despite displaying the expected size. The deviating migratory patterns of Dfr-S*/L* may be due to posttranslational modifications. Of note, cell transfections with *dfr* cDNA only produces the Dfr-S*/L* bands, further indicating that these are indeed isoforms of Dfr (Fig. 1e). Lack of alternative splicing or potential RNA editing proximal to the stop codon was experimentally confirmed by DNA sequencing of a reverse-transcribed mRNA (Additional file 1 a-b). Taken together, these results indicate that SCR of *dfr* occurs to a high degree in vivo.

The first stop codon of *dfr* mRNA is a UAG triplet, which has an intermediate relative potential for readthrough (UGA > UAG > UAA) [8]. The frequency of SCR of *dfr* mRNA may be positively influenced by the presence of a cytosine immediately 3′ of the stop codon (UAG-C) and a predicted RNA:RNA stem loop structure immediately downstream of the stop codon [18]. To experimentally verify SCR of *dfr* mRNA and to determine the amino acid decoded from the UAG stop codon, a plasmid was designed for expression of full-length Dfr, tagged with Myc in the C-terminal end of ORF2 (*dfr-L-Myc*, Fig. 1d). In this way, Myc should only be translated if SCR occurs, and only tag Dfr-L. Compared to untagged *dfr*, S2 cell transfection with *dfr-L-Myc* resulted in a size-shift of Dfr-L*, but not Dfr-S*, when incubating the blot with anti-Dfr-S/L (Fig. 1e, left panel). This indicates that the Myc-tag inclusion has increased the protein size, as expected, which was corroborated by incubation with anti-Myc (Fig. 1e, right panel). To provide further experimental evidence that Dfr undergoes SCR, immunoprecipitation of Dfr-L-Myc and in-gel digestion with chymotrypsin was performed followed by liquid chromatography-coupled tandem mass spectrometry (LC-MS/MS) analysis (Fig. 1d, f, Additional file 2). A MASCOT analysis against a reference *Drosophila* protein database resulted in 15 peptides with sequences uniquely aligning to Dfr (Fig. 1f, Additional file 2 c). Importantly, four peptides matched within the C-terminal extension and one encompassed the first in-frame UAG stop codon, which demonstrates that *dfr* mRNA undergoes SCR (Fig. 1c, Additional file 2 b). The only amino acid incorporation detected at the readthrough stop codon was glutamine, found in two separate peptides with identical sequence but different charges, one of which produced a significant ion score (45.4). This indicates that the UAG codon was interpreted as a CAG codon, as AAG and GAG would be translated into lysine and glutamic acid respectively. Besides Dfr, sequences matching six other *Drosophila* proteins were detected. These were typically high abundance proteins, like Myosin 31DF and Myo61F, suggesting a degree of impurities typical for this sort of pull-out assay. A second MASCOT analysis was performed using the Dfr-L-Myc sequence as reference, resulting in two peptides found to match the Myc-tag sequence (ISEEDLNHRST, Score = 68; GGAGGAGGEKGGRADPAFLY, Score = 11), the latter scoring below the significance threshold but spanning the expected Dfr-L-Myc fusion. We conclude that the UAG stop codon in *dfr* mRNA can be used as a template for tRNA^gln^ base pairing and incorporation of glutamine.

### The C-terminal extension of Dfr is evolutionarily conserved in Diptera

To investigate the evolutionary conservation of the Dfr C-terminal extension, we performed a phylogenetic analysis using amino acid sequences from ORF1 and putative ORF2 independently (Fig. 2). As outliers, three non-dipteran species were included (*Tribolium castaneum*, *Rodnius prolixus*, and *Heliconius melpomene*), all of which produced a short putative ORF2. In common for ORF1 and 2, the resulting trees displayed similar patterns of divergence, although *D. melanogaster* ORF2 was more distant to members of Culicidae and outliers (Fig. 2a, b). Within closely related dipteran species including *Drosophila*, *Lucilia*, and *Glossina*, ORF2 was conserved (> 87% sequence coverage and > 65% identity), suggesting that a potential biological role of the extended form may also be preserved (Fig. 2c). Less conservation was found among Culicidae when compared to *D. melanogaster* (> 43% sequence coverage; 17.6–18.4% identity). Interestingly, *dfr* SCR has been proposed to occur in the malaria mosquito *Anopheles gambiae* as well, despite the low degree of sequence identity in ORF2 compared to *D. melanogaster* [18]. Searches for putative functional domains with InterPro and ELM within ORF2 did not provide any high fidelity hits. Similar to ORF1, *D. melanogaster* ORF2 has a high proportion of the amino acids His, Ala, and Pro (Figs. 1f and 2c). Conversely, Gln, Ser, and Asn are more frequent in ORF2, which also contains stretches of Gln, Asn, and His/Pro. Overall, similar compositions were observed in ORF2 of other species compared. Multiple sequence alignment of ORF2, indicated that the first few bases proximal to the stop codon as well as a region near the C-terminal end are preserved from *Drosophila* to *Culicidae*, (Fig. 2d). Stretches of amino acid repeats were prominent in all species, but with a low degree of alignment between mosquitoes and flies. Such low complexity regions are frequently observed in *trans-*activation domains (tADs) of eukaryotic transcription factors [37, 38], and it also suggests the presence of intrinsically disordered regions (IDRs). This was confirmed using IUPred and PONDR analytical tools, which predicted that the entire C-terminal extension, apart from the C-terminal end, is disordered (Fig. 2e). Interestingly, the presence of large IDRs was evident in all predicted SCR-derived C-terminal extensions of *dfr/vvl* (Fig. 2e, Additional file 3 a-d). Thus, the general physico-chemical properties of the C-terminal extension may be more relevant than the precise position of specific amino acids for its biological function(s).

### Spatiotemporal regulation of *dfr* stop codon readthrough

We next analyzed the relative expression levels of Dfr-S and Dfr-L isoforms in different tissues and stages of development. Immunostaining using anti-Dfr-S/L and Dfr-L antibodies in parallel revealed that both Dfr isoforms are predominantly nuclear, indicating that SCR does not change the subcellular localization of Dfr (Fig. 3a–i). The PG of all three larval instars stained intensively with both antibodies (Fig. 3a, d, g) as well as the ring gland in late-stage embryos (Additional file 4 f and i), indicating prominent SCR. This was confirmed in extracts of brain/ring gland complexes (BRGCs) where the relative abundance of Dfr-L (Dfr-L/L* relative to Dfr-S/S*) reached 47% in females and 43% in males, demonstrating a very high degree of SCR (Fig. 3j). A developmental profile of BRGCs showed that SCR was frequent in early L3 larvae and then declined (Fig. 3k, l). A high level of *dfr* SCR was also observed in larval trachea (Fig. 3b, e). Dfr-L immunostaining was further detected in L2 brain and CNS (Fig. 3g), in adult fat body cells and oenocytes (Fig. 3h, i), and in several other larval and adult tissues (Additional file 4). In contrast, Dfr-L was barely detectable in the male ejaculatory duct (Fig. 3f), adult trachea and ureter cells, in which anti-Dfr S/L staining was prominent (Additional file 4 d-e, i [30];). Thus, these tissues primarily express the Dfr-S isoform and seem resistant to *dfr* SCR. This indicates that SCR of *dfr* is a highly regulated process, ranging from 50% in tissues like the larval PG, to tissues with high *dfr* gene expression without prominent SCR, such as the male ejaculatory duct. This underscores that the rate of *dfr* SCR is not simply the result of leaky translational termination. We conclude that *dfr* undergoes SCR in a spatiotemporal manner, suggesting that it is programmed as part of a gene regulatory program.

### Larval to pupal transition is delayed in mutants that cannot produce Dfr-L

**Fig. 4:**
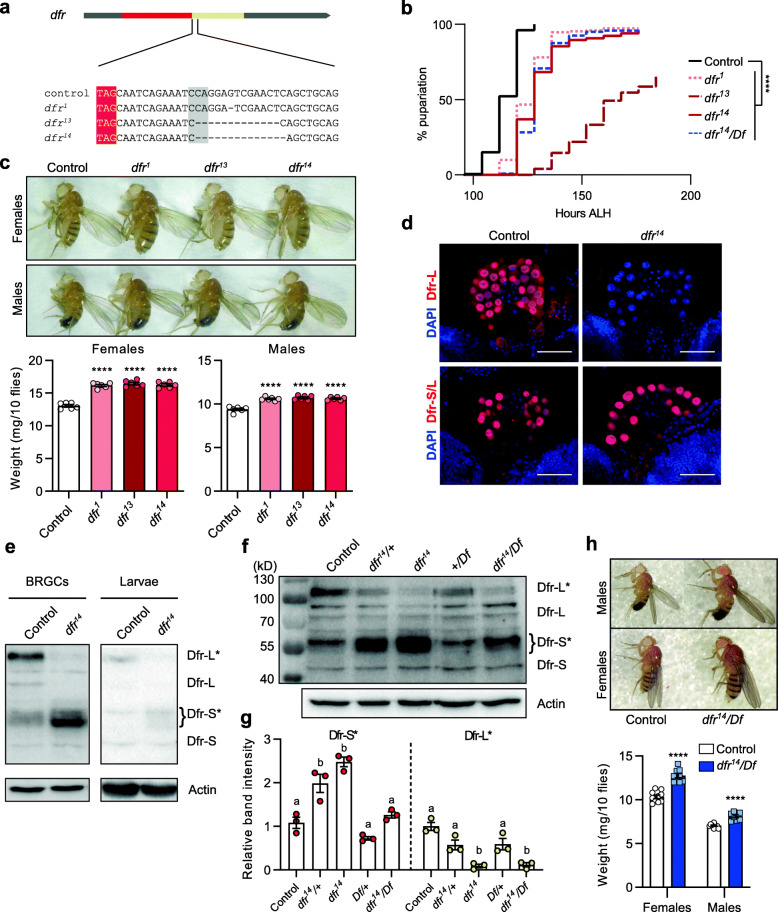
A 14-nt deletion downstream of *dfr* first in-frame stop codon leads to developmental defects. **a** Upper panel, *dfr* gene structure; lower panel, nucleotide sequences of control *w*^*1118*^ and *dfr* mutants. Three different *dfr* mutant strains were isolated, with 1, 13, and 14 nucleotide deletions downstream of the annotated stop codon, and named accordingly. The canonical stop codon is highlighted in red. The protospacer adjacent motif (PAM) sequence targeted for cleavage by the CRISPR/Cas9 system is highlighted in gray. **b** Percentage of larvae undergoing pupariation over time after larval hatching. In parallel, *dfr14/Df* was monitored (*n* = 5–10; *N* = 150–300; p**** < 0.0001; see **g–h**). **c** Upper panel: representative images of male and female adult sizes of control *w*^*1118*^, *dfr*^*1*^, *dfr*^*13*^, and *dfr*^*14*^. Lower panel: quantification of wet fly weight of males and females raised in non-crowded conditions (30 larvae/vial). Data represent mean + SE (*n* = 6). **d** Immunostainings of control and *dfr*^*14*^ BRGCs with anti-Dfr-L (upper panels) and anti-Dfr-S/L (lower panels). Dfr-L was absent in *dfr*^*14*^ prothoracic glands. Scale bars 100 μM. **e** Immunoblots of BRGC extracts (left), and whole larval extracts (right), without fat body and intestine from control and *dfr*^*14*^ larvae, incubated with anti-Dfr-S/L. Actin was used as a loading control. The band corresponding to Dfr-L/L* was barely detectable, while Dfr-S* increased in *dfr*^*14*^ mutants. **f** Representative anti-Dfr-S/L immunoblot prepared from BRGCs of denoted genotypes. Anti-Actin was used as loading control. +, wild type allele; Df, *Df(3 L)*Exel6109. **g** Quantification of normalized band intensities of Dfr-S* and Dfr-L*. Bar plots represents mean + SE from four independent replicates. Distinct lettering indicates significantly different intensities (*p* < 0.05). **h** Left panel: representative images of male and female adult flies of control *w*^*1118*^ and *dfr*^*14*^*/Df.* Right panel: quantification of wet fly weight of males and females (*w*^*1118*^, *n* = 10, *N* = 100; *dfr*^*14*^*/Df*, *n* = 8*, N* = 80; p**** < 0.0001)

To study the function in vivo of Dfr-L, we generated *dfr* mutations using CRISPR/Cas9-mediated genome editing. We isolated three different mutants carrying 1, 13, and 14 bp deletions downstream of the first in-frame stop codon, and designated them *dfr*^*1*^, *dfr*^*13*^, and *dfr*^*14*^ respectively (Fig. 4a). Homozygous larvae of all three mutants displayed developmental delays, requiring between 5.5 and 7.5 days before pupariation, compared to 5 days for control (Fig. 4b). Consequently, *dfr*^*1*^, *dfr*^*13*^, and *dfr*^*14*^ adults were bigger than controls and their measured weight was increased (Fig. 4c). We focused further work on the *dfr*^*14*^ mutant, in which the 14 bp deletion removed part of a predicted RNA hairpin structure just 3′ of the stop codon, as well as causing a frameshift followed by numerous stop codons in the extending reading frame (Fig. 4a). Immunostaining of ring glands and other tissues with anti-Dfr-L did not produce any detectable staining in *dfr*^*14*^, demonstrating that Dfr-L synthesis was impaired by the deletion (Fig. 4d, Additional file 4 i). Conversely, both control and mutant stained positively for anti-Dfr S/L, which suggests that the mutation neither affects *dfr* transcription, nor translation negatively until the first stop codon is reached. The impaired Dfr-L expression in *dfr*^*14*^ BRGCs and whole larvae was confirmed on immunoblots (Fig. 4e). As expected, loss of SCR also ensued a higher relative concentration of Dfr-S, or possibly a severely truncated Dfr-L that would likely act as Dfr-S, as the isoforms are encoded from the same transcript. To be able to analyze Dfr expression in larvae with different copy numbers of *dfr-S* and *dfr-L* encoding capacity, we crossed controls or *dfr*^*14*^ to flies carrying a large deficiency, *Df(3 L)Exel6109* (*Df*), encompassing the *dfr* locus. The band corresponding to Dfr-L* was significantly reduced in homozygous *dfr*^*14*^ and in *dfr*^*14*^/*Df* larval extracts compared to control (Fig. 4f, g). As expected in heterozygous mutants (*dfr*^*14*^*/+* and +/*Df*), Dfr-L* was significantly reduced compared to control but elevated relative to *dfr*^*14*^ and in *dfr*^*14*^/*Df*. The Dfr-S* band intensity increased reciprocally with the loss of Dfr-L-coding alleles. By removing a functional allele (*dfr*^*14*^/*Df*), the expression of Dfr-S* could be reverted to control levels. Importantly, this genotype displayed a retained developmental delay similar to *dfr*^*14*^ (Fig. 4b), as well as increased adult size and weight (Fig. 4 h), which suggests that these phenotypes arise primarily due to loss of Dfr-L* protein rather than an increase in Dfr-S* concentration. From these results, it can be concluded that *dfr* SCR is necessary for correct timing of pupariation and metamorphosis.

### The transcriptome is extensively dysregulated in larvae lacking the Dfr-L isoform

**Fig. 5:**
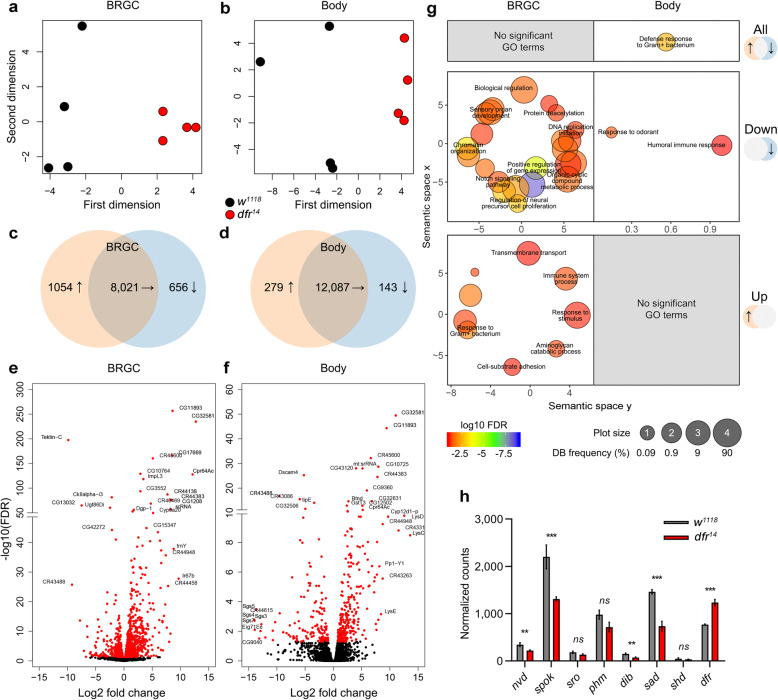
Absence of Dfr-L causes extensive transcriptional changes in the BRGC of wandering larvae. **a–h** RNA-seq analysis of BRGCs or bodies derived from control versus *dfr*^*14*^. Data were generated from all transcripts with a designated FBgn number and expressed in at least one of the groups in respective tissue. Each tissue was analyzed separately. **a, b** Two-dimensional scatterplot depicting leading log2 expression differences between the transcriptomes of controls versus *dfr*^*14*^ derived from either BRGC (**a**) or body (**b**). **c, d** Venn diagrams of unaltered, increased or decreased transcript levels in *dfr*^*14*^ (FDR < 0.05) in BRGC (**c**) and body (**d**). For full list of DEGs, see Additional File 5. **e, f** Vulcano plots of all genes in BRGC (**e**) and body (**f**), expressed in at least two out of the four groups. Differentially expressed transcripts are highlighted in red. **g** Gene ontology (GO) analysis was performed using GOrilla (terms with an FDR < 0.1 were considered) and summarized in REVIGO to remove redundant terms (dispensability > 0.5). Separate analyses were performed using either all (top panels), downregulated (middle panels), or upregulated (bottom panels) hits from respective tissue. Circle sizes represent GO term frequency (in log10-scale) in the underlying database, e.g., a small circle depicts a more specific term. Circle color and scale bar reflects log10 FDR value. Scatterplot axes refer to semantic similarities between GO terms within a two-dimensional space (the values have no intrinsic meaning per se). For ease of viewing, the dispensability threshold was set to < 0.2 for spelled out GO terms. For the complete list of GO terms, see Additional file 6. **h** Comparative bar graph of BRGC expression of Ecdysone biosynthesis genes and *dfr*. Asterisks indicate differential expression between groups (FDR < 0.01). *shade (shd)* was included as a negative control since it is not normally expressed in the BRGC. For the complete list of ecdysone-associated genes, see Additional file 7

We reasoned that the C-terminal extension might provide Dfr-L with unique features in transcriptional regulation. To investigate this, RNA-seq analysis was applied to compare the transcriptome profiles in BRGCs (where SCR is very prominent) and in body tissues, separately, from *dfr*^*14*^ third instar wandering larvae to those of controls. A multidimensional scaling (MDS) analysis was performed based on the leading fold changes to compare Euclidean distances between replicates according to the first two dimensions (*x*-axis, first dimension, *y*-axis second dimension; Fig. 5a, b). This indicated a genotype-specific separation along the first dimension in both tissues. The total number of differentially expressed targets (FDR < 0.05) with a designated flybase gene number (FBgn; from hereon referred to as differentially expressed genes [DEGs]) was clearly larger in the BRGC than in the body (Fig. 5c, d and Additional file 5), correlating with the high rate of *dfr* SCR in this tissue. Several DEGs were strongly affected in the mutant, e.g., 53 in the BRGC and 82 in body had a log2 ± fold change > 5 (Fig. 5e, f). Gene ontology (GO) enrichment analysis of terms associated with biological processes revealed that the bulk of significant terms were linked to DEGs with reduced expression (down) in the BRGC of *dfr*^*14*^ (Fig. 5g), indicating that SCR of *dfr* plays a role upstream of the expression of these genes. These enriched terms encompassed diverse processes such as “positive regulation of gene expression,” “DNA replication initiation,” “protein deacetylation,” “sensory organ development,” “chromatin organization,” and “Notch-signaling,” to name a few (see Additional file 6 for the full list). For DEGs with increased expression in the BRGC in the *dfr*^*14*^ mutant, enrichment and diversity were lower, but revealed some diverse processes. In the body, only a few processes, associated with immunity and odor sensing, were significantly downregulated. Enrichment analysis was also performed on terms related to molecular function (Additional file 6). In the BRGC and associated with DEGs with decreased expression, all enriched terms were related to DNA binding functions, including “transcription factor/cofactor activity” and “chromatin binding”. This suggests that the altered Dfr isoform ratio in *Dfr*^*14*^ has broad downstream effects on the transcriptome by affecting the expression of additional transcriptional regulators.

It has earlier been shown using RNA interference (RNAi) that Dfr is involved in regulation of the ecdysone biosynthesis genes expressed in the prothoracic gland (PG, see below), *neverland (nvd)*, *spookier (spok)*, *shroud (sro)*, *phantom (phm)*, *disembodied (dib)*, and *shadow (sad)* [35, 36]. In these studies, the discrete roles of Dfr-S and Dfr-L were not considered as the RNAi targeted both isoforms. Here, we found that expression of *nvd*, *spok*, *dib*, and *sad* is reduced in the BRGC of *dfr*^*14*^ (Fig. 5h). This indicates that SCR of *dfr* is required for normal expression levels of these genes and, consequently, for steroidogenesis. Of note, expression levels of *dfr* mRNA per se was slightly but significantly increased, possibly reflecting *dfr* autoregulation [30, 39]. We therefore ruled out that the observed effects on ecdysone biogenesis genes were caused by impaired *dfr* mRNA expression.

Defective ecdysone levels affect the temporal expression of ecdysone-responsive genes. Despite lacking the temporal aspect, the RNA-seq data revealed abolished expression of members of the salivary gland secretion family (*Sgs3*, *Sgs4*, *Sgs5*, *Sgs7*, *Sgs8*) and the ecdysone-inducible gene *Eig71Ee* in the body of *dfr*^*14*^ (Fig. 5f and Additional file 7). In summary, these findings show that the inability of *dfr*^*14*^ mutants to produce the Dfr-L isoform by SCR has extensive effects on the transcriptome of wandering larvae.

### Expression of the steroidogenic enzymes Nvd, Spok, Dib, and Sad is modulated by *dfr* SCR

**Fig. 6:**
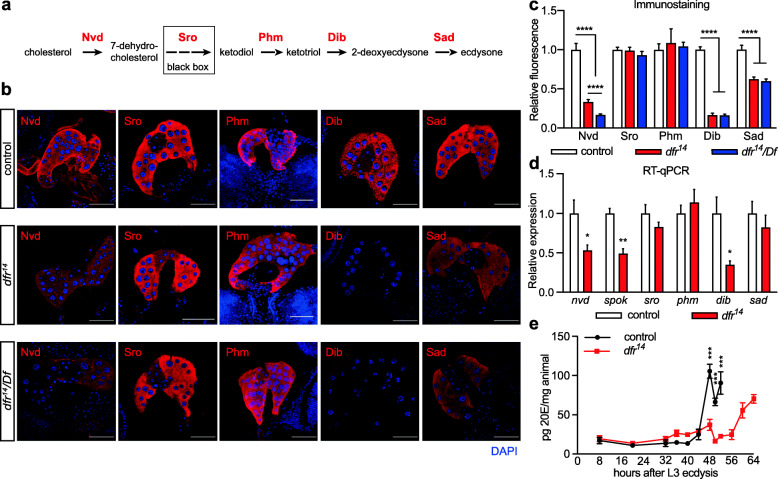
The expression of Nvd, Spok, Dib and Sad is compromised in the *dfr*^*14*^ mutant. **a** Ecdysone biosynthesis steps in the *Drosophila* prothoracic gland. Steroidogenic enzymes Nvd, Sro, Phm, Dib, and Sad are sequentially required to convert cholesterol to ecdysone. **b** Immunostaining of control (upper panels), homozygous *dfr*^*14*^ (middle panels), and hemizygous *dfr*^*14*^ */Df* (lower panels) BRGCs for the steroidogenic enzymes Nvd, Sro, Phm, Dib, and Sad. The immunostaining of the enzymes is shown in red, DAPI staining in blue. Scale bars 50 μm, except for the images stained for Sad (100 μm in these images). **c** Quantification of relative fluorescence intensity in **b**. The expression of Nvd, Dib, and Sad was significantly reduced in *dfr*^*14*^ and *dfr*^*14*^ */Df,* compared to control. **d** RT-qPCR results of the steroidogenic genes. Transcript levels of *nvd*, *spok*, and *dib* were significantly decreased in *dfr*^*14*^ (*n* = 5; Q* < 0.05, Q** < 0.01). **e** Ecdysone concentrations were quantified in a 2-, 4-, or 12-h time window from early to late L3. *n* = 4 for each time point. For comparison, 40 h ALE is approximately equal to 88 h ALH in Fig. 3k, l. Asterisks denote significant differences at each time point (*n* = 4; Q*** < 0.0001)

To gain further mechanistic understanding of how loss of *dfr* SCR impairs ecdysone biosynthesis and how it feeds into the timing of developmental transitions, we focused the subsequent investigations in this direction. In *Drosophila*, the neuroendocrine organs corpora allatum (CA), PG, and corpora cardiaca (CC) are fused into a compound structure, the ring gland (RG), which is attached to the brain (Additional file 8 A). To visualize the three-dimensional (3D) structure of this endocrine organ, we performed 3D reconstructions based on a confocal stack of a BRGC (Additional files 8 and 9). The PG is composed of the large ring gland lateral cells; CA cells are smaller, medial in the RG. The PG is the site for the ecdysone biosynthetic pathway with expression of all the enzymes required for the biosynthesis from cholesterol to ecdysone (Fig. 6a). The last step of the ecdysone biosynthesis pathway, the conversion of ecdysone to the bioactive 20-hydroxyecdysone, takes place in peripheral tissues and is catalyzed by the enzyme Shade (Shd) [40]. Consequently, *shd* expression was not changed in *dfr*^*14*^ mutant BRGCs (Fig. 5h)

In line with the transcriptome data, immunostaining of homozygous *dfr*^*14*^ and *dfr*^*14*^ */Df* mutant BRGCs showed that the expression of the steroidogenic enzymes Nvd, Dib, and Sad, but not Phm and Sro, was significantly decreased in both mutant genotypes compared with control (Fig. 6b, c). This was also confirmed by quantitative reverse transcriptase-PCR (qRT-PCR) of BRGCs (Fig. 6d), validating the RNA-seq data (Fig. 5h), including also the significant reduction of *spok* mRNA. To explore the functional importance of *dfr* SCR for temporal ecdysone production, we performed kinetic profiling of 20E titers in *dfr*^*14*^ and control larvae after L3 ecdysis (AL3). As expected, control larvae showed a peak of 20E prior to pupariation, around 48 h AL3. Importantly, the time period of strong rise in ecdysone levels (40–48 h AL3) refers approximately to 88–96 h AEL (Fig. 3k, l), when Dfr-L* levels are high in the BRGC. In *dfr*^*14*^ mutant larvae, however, only a minor peak was observed at this time point and 20E titers remained low until around 60 h AL3 (Fig. 6e). This indicates that *dfr* SCR is required to properly time the ecdysone titer peak necessary for pupariation.

### Overexpression of either Dfr-S or Dfr-L in the prothoracic gland causes developmental arrest

**Fig. 7:**
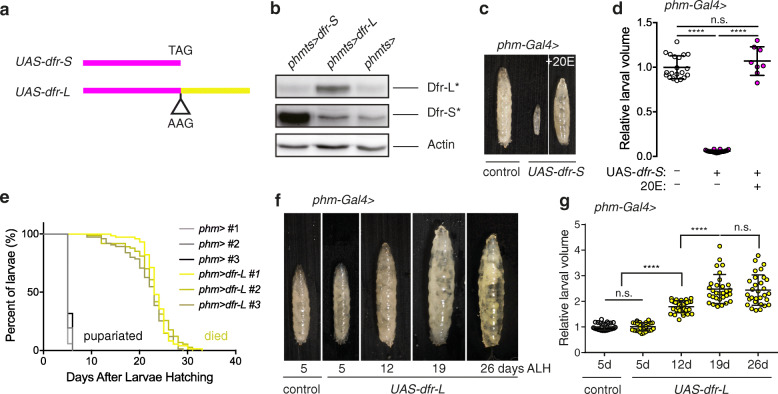
Dfr-S and Dfr-L overexpression causes arrest at different developmental stages. **a** Schematic drawing of expression constructs for Dfr-S and Dfr-L respectively. A point mutation was introduced to change the stop codon TAG to a lysine codon, AAG. **b** Overexpression of *UAS-dfr-S* and *UAS-dfr-L* in larval PG using a temperature-sensitive *Phm-Gal4* driver (*Phm-Gal4[ts]).* Immunoblot using anti-Dfr-S/L. Proteins were extracted from three BRGCs for each genotype. Actin was used as loading control. **c–g** Overexpression of *UAS-dfr-S* and *UAS-dfr-L* in larval PG using the *Phm-Gal4* driver. **c, d** Overexpression of Dfr-S in the PG led to arrest at first larval instar (L1). The developmental arrest was partially rescued by 20E feeding (+20E). **d** Quantification of the relative larval volume. **e–g** Overexpression of Dfr-S in the PG. **e** Percentage of individuals in larval stage until entering pupariation (control, blue) or eventually succumbing to death (Dfr-L overexpressing larvae). Control larvae (*phm-Gal4>*) pupariated at around 5–6 days after larval hatching. *phm-Gal4>UAS-dfr-L* led to L3 arrest. The larvae were not able to pupariate and died in the end. **f** Representative images of larvae at indicated days ALH. *phm-Gal4>UAS-dfr-L* expression resulted in giant larvae. **g** Quantification of the relative larval volume in panel **f**. There was no significant difference in larval volume between *phm-Gal4>UAS-dfr-L* and control larvae at 5 days ALH. The *phm-Gal4 > UAS-dfr-L* larval volume increased with time

To study Dfr isoforms independently, we attempted to create a Dfr-S mutant, in addition to *dfr*^*14*^, by mutating the wobbling base of the first stop codon. We consistently failed at detecting any positive heterozygotes for the mutation in the resulting F1 generation, suggesting that such a mutation is dominant lethal. Instead, we analyzed the effects in vivo of targeted Dfr-S and Dfr-L overexpression, using independent *UAS-dfr-S* and *UAS-dfr-L* transgenic flies crossed with a temperature-sensitive Gal4 driver, *phm-Gal4*^*ts*^ (*tub-Gal80*^*ts*^*; phm-Gal4*), to drive expression of the two isoforms in the PG at specific times of development. The *dfr-S* construct carries sequences encoding ORF1 only (Figs. 1a and 7a). The *dfr-L* construct was created by introducing a point mutation, converting the first TAG stop codon to AAG, thereby acting as an obligatory ORF1-ORF2 fusion transgene (Fig. 7a). Overexpression of each isoform was confirmed in extracts from BRGCs dissected from synchronized late L3 larvae (Fig. 7b).

To our surprise, overexpression of *dfr-S* or *dfr-L* did not promote premature development. Instead, these phenocopied loss-of-function mutations in ecdysone biosynthesis genes, but at distinct developmental stages. Overexpression of *dfr-S* in the PG, led to developmental arrest at first larval instar (L1), a characteristic phenotype due to lack of ecdysone production, and also the phenotype of *dfr*-RNAi [35]. Partial rescue was observed upon 20E provision in the diet, as larvae developed into L2, but not L3 or pupae (Fig. 7c, d), indicating that these larvae did not produce enough ecdysone. Overexpression of *dfr-L* in the PG also led to developmental arrest, but in the L3 stage (Fig. 7e–g), indicating that the ecdysone titers were appropriate in L1-L2 larvae, but not for pupariation. These L3 larvae continued to feed for more than 5 days, thereby gaining weight and volume (Fig. 7f, g), and stayed at juvenile stage for up to 1 month until death (Fig. 7e). Since there was no difference in volume between control and *dfr-L* overexpression larvae at day 5 after larval hatching (ALH), we concluded that larval growth rate was not affected per se and that the primary phenotype is the inability to pupariate. Furthermore, *dfr-L* overexpression using a weaker and highly PG-specific driver, *spookier-Gal4 (spok-Gal4*) [41, 42], did not block pupariation, but caused a significant delay in pupariation onset (Additional file 10 a), indicating that also moderate overexpression of *dfr-L* in the PG affects developmental progression.

The delayed onset of pupariation also led to an increase in pupal volume (Additional file 10 b-c). An attempt to overexpress *dfr-L* in *dfr*^*14*^ mutant background did not rescue the mutant phenotype; instead, these pupae melanized and shrunk inside the pupal case and died. Of note, this genotype is expected to express elevated levels of both Dfr-S (from the *dfr*^*14*^ allele as in Fig. 4h) and Dfr-L, suggesting that the expression of both Dfr isoforms needs to be tightly regulated to maintain normal development. Further analysis confirmed that overexpression of *dfr-S*, as well as *dfr-*RNAi, had a strong negative effect on *nvd*, *phm*, *dib*, *spok*, and *sad* mRNA expression levels, while *dfr-L* overexpression showed less dramatic effects (Additional file 11 a). A possible explanation for how overexpression of Dfr isoforms can cause similar effects as RNAi on target genes is that balanced concentrations of Dfr-S and Dfr-L may be important for the formation of transcription initiation complexes. Overexpression of one isoform relative to other regulatory factors may disrupt such complex formation and reduce target gene expression, as illustrated schematically in Additional file 11 c. Furthermore, transfection experiments in S2 cells (Additional File 11 b), indicated that Dfr-L is sensitive to high overexpression of Dfr-S, as Dfr-L abundance decreased abruptly when the relative levels of Dfr-S to Dfr-L increased above 1:1. Thus, balanced levels of Dfr-S and Dfr-L are important for appropriate regulation of the ecdysone biosynthesis genes, and their relative abundance may be fine-tuned during development by controlling the level of *dfr* SCR.

### Clonal overexpression of Dfr-S depletes Dfr-L, leading to loss of ecdysone biosynthesis gene expression in a cell-autonomous manner

**Fig. 8:**
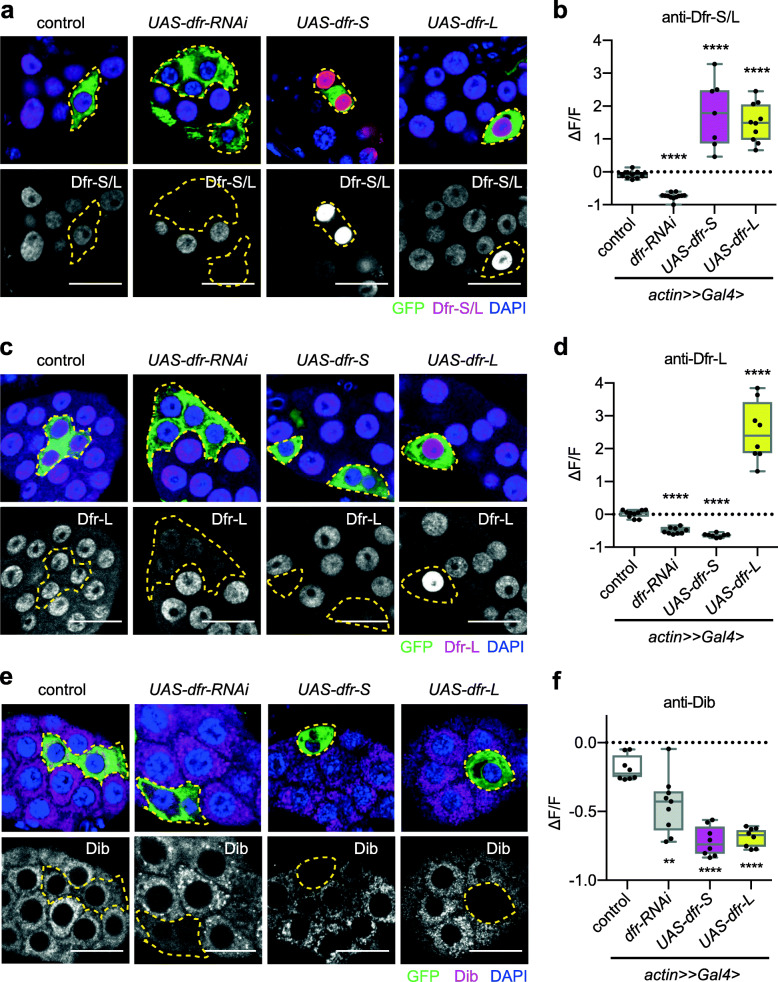
Effects of isoform-specific overexpression of Dfr in the prothoracic gland. **a–f** Confocal stacks of prothoracic glands (PGs) carrying GFP-labelled flp-out clones that express different transgenes (control, *UAS-dfr-RNAi*, *UAS-dfr-S*, and *UAS-dfr-L*). Flp recombinase activity was induced by heatshock at L1 stage, which stochastically removed a stop cassette downstream of the *Actin* promoter in the tissue. Thereby, *Actin-Gal4* expression was activated, which directed the expression of the target transgenes within the clone, including marking the clones with GFP. The PGs were stained with anti-, Dfr-S/L (**a**), Dfr-L (**c**), or Dib (**e**). Clones are outlined with yellow dashed lines. **a, c** Upper panels: anti-Dfr-S/L staining is shown in red, GFP in green, DAPI stains nuclei in blue. Lower panels: Dfr-S/L staining shown in gray. **b** Quantification of relative fluorescence changes in the clones from panel **a**. ∆F/ F measures the change of fluorescence intensity between a clone cell and a neighbor cell. **d** Quantification of relative fluorescence changes in the clones from panel **c**. **e** Upper panels: Anti-Dib staining is shown in magenta, GFP in green, DAPI stains nuclei in blue. Lower panels: anti-Dib staining is shown in gray. Scale bars, 25 μM. **f** Quantification of the relative fluorescence changes in panel **e**

To decipher the isoform-specific regulatory effects on steroidogenesis enzyme expression in vivo, mosaic clonal analyses were performed. We first analyzed Dfr immunostaining in GFP-marked *flp-*out clones overexpressing *dfr-S* or *dfr-L*. The nuclear anti-Dfr S/L fluorescence intensity was significantly increased, while it was lost in *dfr-RNAi* clones, as expected (Fig. 8a, b). Staining of *dfr-RNAi* and *dfr-L* clones with anti-Dfr-L showed a similar pattern (Fig. 8c, d). Surprisingly, in *dfr-S* overexpressing clones, the anti-Dfr-L signal was gone, indicating that *dfr-S* overexpression led to depletion of Dfr-L in the PG (Fig. 8c, d), corroborating the results from S2 cell transfections (Additional File 11 b). Using the same strategy, immunostainings of Nvd, Phm, Dib, and Sad showed that reducing *dfr* expression by RNAi led to a significant reduction of all four proteins in GFP-labelled *flp*-out clones compared to control clones, confirming the critical role of *dfr* in activation of *nvd*, *phm*, *dib*, and *sad* genes (Fig. 8e, f and Additional file 12 a-f) [35]. Overexpression of either *dfr-S* or *dfr-L*, also suppressed Nvd, Phm, Dib, and Sad proteins expression in the PG clones (Fig. 8e, f and Additional file 12 a-f), strengthening the conclusion that overexpression of *dfr-S* or *dfr-L* leads to ecdysone defects (see also Additional file 11 c). Taken together, the marked decrease of several of the ecdysone biosynthesis genes after overexpression of *dfr-S* and *dfr-L* provides a likely explanation to the developmental arrest phenotypes presented in Fig. 7. It further highlights the complex regulation of ecdysone biosynthesis genes where the regulatory roles of the discrete Dfr isoforms may depend on additional factors.

### Dfr-L and Molting defective (Mld) synergistically activate transcription of ecdysone biosynthesis genes

**Fig. 9:**
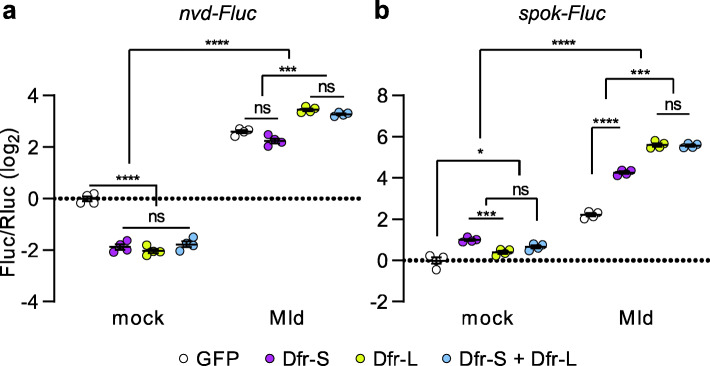
SCR-dependent extension of Dfr-L acts as a transcriptional activator when coexpressed with Molting defective. **a, b** Transcriptional reporter assays with *nvd-Fluc* (**a**) and *spok-Fluc* (**b**) expression in response to expression of Dfr-S, Dfr-L, and Mld independently, and in combination in *Drosophila* S2 cells. Cells transfected with a GFP expression plasmid were used as a negative control. *Y*-axis shows the luminescence of firefly luciferase over renilla luciferase (Fluc/Rluc). *n* = 4. *p < 0.05; ***p < 0.001; ****p < 0.0001

Since attempts to both increase and reduce the expression of Dfr isoforms resulted in similar phenotypes, we hypothesized that their regulatory features may depend on interactions with additional transcription factors. As proof of concept, we performed firefly luciferase (Fluc) reporter assays in *Drosophila* S2 cell cultures, focusing on two of the ecdysone biosynthesis genes, *nvd* and *spok*, whose expression was hampered in *dfr*^*14*^ BRGCs (Figs. 5h and 6d). A well-characterized regulator of *nvd* and *spok* is the zinc-finger transcription factor Molting defective (Mld) [35, 43, 44]. These genes also contain putative binding sites for Dfr [35]. In line with transcriptional data (Additional file 11 a), transfection with *dfr-S*, *dfr-L*, or both, repressed *nvd-Fluc* (Fig. 9a). Conversely, expression of *spok-*Fluc was slightly but significantly enhanced, suggesting that Dfr regulates *nvd* and *spok* differently (Fig. 9b). In accordance with the aforementioned studies, expression of Mld resulted in a roughly 5-fold increased signal from both *nvd-Fluc* and *spok-Fluc* (Fig. 9a, b). Strikingly, cotransfection with *dfr-L* and *mld* expression plasmids synergistically activated both *nvd-Fluc* and *spok-Fluc* reporters (approximately 10-fold and 50-fold, respectively), indicating a coordinated role of Dfr-L and Mld in the regulation of *nvd* and *spok* expression (see Additional file 11 c for a schematic illustration). On the contrary, cotransfection with *dfr-S* and *mld* did not affect *nvd-Fluc* expression significantly compared to *mld* transfection alone, whereas *spok-Fluc* was activated but to a less degree than *dfr-L* and *mld* (Fig. 9a, b). Neither *nvd-Fluc* nor *spok-Fluc* signal was altered by the combination of *dfr-S*, *dfr-L*, and *mld* compared to *dfr-L* and *mld*, suggesting that the two Dfr isoforms do not negatively impact the activity of the other, when expressed at equal levels. In conclusion, with regard to *nvd* and *spok*, full *trans-*activation capacity of Dfr was dependent on the SCR-dependent C-terminal extension.

## Discussion

**Fig. 10:**
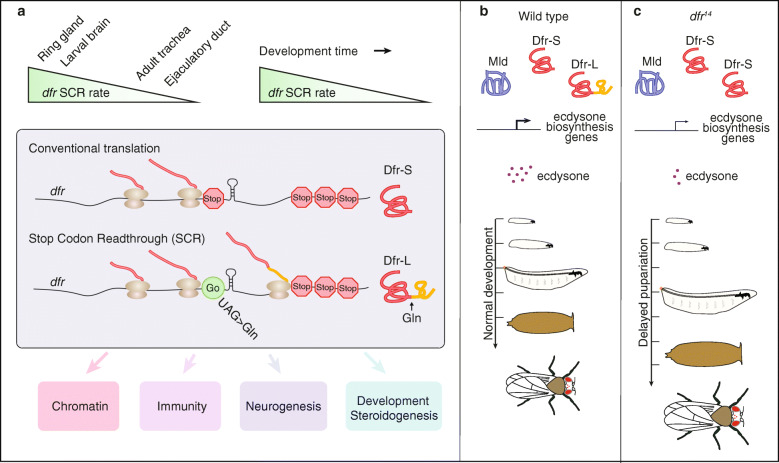
Translational stop codon readthrough alters the output of Dfr as a transcriptional activator, and modulates hormone-regulated timing of developmental transitions. **a** Top: SCR of *dfr* mRNA varies in a spatiotemporal manner with high rates in larval brain and ring gland and low in adult trachea and ejaculatory duct. All these tissues confer high level expression of *dfr* mRNA (Fig. 3), indicating tissue-specific regulation of SCR. *dfr* SCR is prominent in brain and ring gland during early stages of larval development, then ceases with larval developmental time (Fig. 4), indicating that SCR is temporally controlled. Middle: Conventional translation of *dfr* mRNA ends at the first canonical stop codon (Stop), producing Dfr-S with 427 amino acids (Fig. 1). Translational SCR of the same *dfr* mRNA continues past the first stop codon by incorporation of a glutamine, and ends at the 2nd stop, producing Dfr-L with 713 amino acids (Fig. 1a). A predicted RNA hairpin structure just 3′ of the first stop codon [18] is likely to be involved in the readthrough mechanism. Bottom: The C-terminal extension of Dfr-L alters the transcriptional output and changes expression of genes involved in downstream biological processes (boxed), as shown by RNA-seq analyses of the transcriptome of *dfr*^*14*^ mutant larvae *vs* controls (Fig. 5). Loss of *dfr* SCR as, in *dfr*^*14*^ mutant larvae, negatively affects steroidogenesis. **b, c** Model of *dfr* SCR-dependent modulation of ecdysone-regulated developmental transitions. **b** Normal expression of the ecdysone biosynthetic pathway genes requires both Dfr-S, Dfr-L and Mld (Fig. 6). Dfr-L and Mld activate *nvd* and *spok* expression in a synergistic manner (Fig. 9), contributing to high ecdysone titers. This ensures correct timing of developmental progression from larval to adult stages. **c** Elimination of *dfr* SCR and production of Dfr-L (e.g., in the *dfr*^*14*^ mutant) compromises ecdysone biosynthesis, causing prolonged larval development, delayed pupariation, and increased adult size (Figs. 4 and 6). Thus, *dfr* SCR modulates hormone-regulated timing of developmental transitions

Programmed, alternative decoding of the genome, such as SCR and translational frameshifting have recently gotten increased attention through comparative genomics analyses and ribosome profiling experiments, indicating that alternative coding is pervasive and evolutionarily conserved [2, 17–19, 45, 46]. In the present study, we provide several lines of evidence to show that *dfr* mRNA undergoes SCR in *Drosophila*. Firstly, the pull-down of a Dfr-L-Myc fusion protein confirmed that Myc was properly translated as a result of SRC of *dfr* mRNA. Secondly, the mass spectrometry identified peptides that matched the C-terminal extension sequence of Dfr. Thirdly, we show that the stop codon UAG was decoded as glutamine. Lastly, immunostaining in larvae and adults with an antibody that recognizes the Dfr C-terminal extension confirmed SCR of *dfr* mRNA in vivo. Strikingly, the readthrough rate of *dfr* is as high as 50% in certain tissues during specific stages of development, indicating that *dfr* SCR is a regulated event with functional consequences (Fig. 10a). We also provide mechanistic understanding to how *dfr* SCR modulates steroidogenesis and how this controls developmental timing of pupariation and metamorphosis (Figs. 6, 7, 8 and 9).

A few studies using ribosomal protection assays in insects and human cells have shown that the rate of SCR differs between tissues and cell types, indicating that SCR is a programmed and regulated process [19, 26, 46]. However, a clear link between the ribosomal profiling data and a functional importance in vivo of SCR has essentially been lacking in metazoans, and it has been argued that SCR is generally nonadaptive [47]. Our work shows that *dfr* SCR differed in a stage- and tissue-specific manner (Fig. 3), strongly indicating the involvement of *trans*-acting factors, such as protein, RNA, or other molecules, interacting with *cis-*acting elements in the affected genes. To identify such *cis*- and *trans-*acting molecules and to elucidate the underlying mechanisms of this regulation will be an important undertaking in future work.

It was recently reported that *Drosophila kelch* mRNA, one of the pioneer SCR candidates [21], confer higher rate of SCR in larval and adult central nervous system tissues compared to some other larval and adult tissues [48]. Surprisingly, there was no SCR of *kelch* mRNA in the PG, albeit a prominent mRNA expression level. This supports that the high rate of *dfr* SCR in the PG is not a result of translational leakage in this endocrine tissue. Three additional *Drosophila* mRNAs (*sponge*, *Ptp10D*, and *hdc*) also granted higher rate of SCR in adult or larval brain tissues, but not in ovaries [48]. This encouraged us to analyze published ribosome profiling datasets and compare the ribosomal occupancy over *dfr* ORF2 between embryos and adult tissues. While there is no or little indication of ribosomal occupancy over ORF2 in embryo tissues, where *dfr* mRNA is highly abundant, there is, however, a prominent occupancy signal in data from adult head tissues, when analyzed using the Ribosome Profiling data base (RPFdb) version 2.0 [49, 50]. Thus, several independent reports support that brain and nervous system tissues are prone to regulated SCR with Dfr seemingly being one of the targets.

Alternative decoding of factors that regulate gene expression could consequentially result in broad changes in downstream processes. For example, SCR of the mammalian gene for Argonaute1 (Ago1) produces the Ago1x isoform, which acts as a competitive inhibitor of the miRNA pathway, leading to increased global translation as a result of SCR [12]. In the present work, a deletion that abolished the C-terminal extension had strong effects on the transcriptional profile of *dfr*^*14*^ mutant larvae, with pronounced effects on genes involved in gene expression, neural proliferation, sensory organ development, and immune system processes. This together with the hampered ecdysone production and developmental delays of *dfr*^*14*^ mutant larvae demonstrates the importance of SCR and suggests that the C-terminally extended Dfr-L isoform play specific roles in vivo. Importantly, Dfr-L activated *nvd-Fluc* and *spok-Fluc* reporters in a synergistic manner together with Mld. Thus, SCR of *dfr* mRNA switches the regulatory output of Dfr, altering its capacity to control the expression of steroidogenic enzymes (Fig. 10a, b). When this SRC-derived regulatory switch is eliminated, as in the *dfr*^*14*^ mutant, ecdysone biosynthesis is impaired and the developmental transitions between different life-cycle stages are delayed (Fig. 10c).

The first step in ecdysone production is the conversion of dietary cholesterol to 7-dehydrocholesterol (7 DC), regulated by Nvd. Remarkably, *nvd* and *spok* are located in the pericentromeric regions thought to form constitutive heterochromatin [51–53]. Expression of heterochromatic genes has been suggested to require epigenetic regulators that control heterochromatic silencing, for example HP1a, and other chromatin remodeling complexes [54]. In this context, it is interesting to note that the transcriptome analysis of the *dfr*^*14*^ mutant lacking Dfr-L revealed that expression of genes involved in biological processes defined as “chromatin organization,” “protein deacetylation,” and “positive regulation of gene expression” were reduced in *dfr*^*14*^ mutant BRGCs.

Interestingly, the extension contains several low complexity regions, together constituting an intrinsically disordered region (IDR) (Fig. 2c, e). Regions enriched for individual amino acids including glutamine, asparagine, histidine, serine, proline, and alanine are well known to be abundant in different classes of tADs [38]. Similar amino acid composition was also evident in the predicted C-terminal extensions of dipteran Dfr/Vvl proteins (Fig. 2c), suggesting that the composition and physico-chemical properties of Dfr C-terminal extensions defines additional properties rather than the primary amino acid sequence per se*.* The presence of glutamine-rich regions is especially intriguing since this feature has repeatedly been connected to tADs [55, 56].

Low complexity regions and IDRs have recently been linked to liquid-liquid phase separation of transcription regulatory complexes [57, 58]. In a computational analysis of *Drosophila melanogaster* SCR candidate proteins, it was found that the C-terminal extensions were significantly enriched in disordered and low complexity regions [26] raising the possibility that these in fact constitute regulatory entities that are added as C-terminal extensions through SCR. We suggest that Dfr-L with its C-terminal extension may play a role in liquid-phase condensate formation, as illustrated in Additional file 11 c. In this model, Dfr-L would support the creation of a transcription initiation complex together with Dfr-S, Mld, and other regulatory factors, promoting appropriate activation of the target genes. For transcriptional regulators like Dfr, for which the SCR is regulated in a spatiotemporal manner, the addition of an IDR/tAD to its C-terminus may thereby have a major impact on a number of cellular and developmental processes. We conclude that SCR of regulatory proteins may play a more prominent role in controlling biological processes than previously anticipated.

## Conclusions

Translational SCR of the POU/Oct transcription factor Dfr generates an evolutionarily conserved C-terminal extension that boosts the capacity of Dfr as a transcriptional regulator. SCR of Dfr takes place in a spatiotemporal manner, strongly indicating that it is genetically preprogrammed. Elimination of the C-terminal extension causes extensive transcriptome alterations of many biological processes, including delayed steroid hormone biosynthesis and subsequent developmental aberrations. Thus, this study demonstrates how SCR of a transcription factor can act as a developmental switch, feeding into the timing of developmental transitions. These findings indicate that translational readthrough may serve as an important regulatory mechanism of many cellular and developmental processes in a spatiotemporal manner. In addition, increased understanding of programmed SCR may open new routes to treat human diseases caused by premature termination codons, which would be of great medical importance.

## Methods

### Fly stocks

Flies were maintained on potato medium [59] at 25 °C unless otherwise indicated with a 12 h light 12 h dark cycle. The *w*^*1118*^, *dfr* deficiency line Df(3 L)Exel6109 (BL7588), *Aug-Gal4* (BL30137), *tub-Gal80ts* (BL7019), *UAS-mCherry* (BL38425), and *vasa::Cas9* (BL51323) were obtained from Bloomington Drosophila Stock Center (BDSC). The *UAS-dfr-S* and *UAS-dfr-L* transgenic lines are described below, the *UAS-dfr-RNAi* line was provided by Sarah Certel and expresses a double-stranded RNA covering nt 517-1308 of *dfr* mRNA [29]; *phm-Gal4* by Kim Rewitz, and *spok-Gal4.1.45* by Michael B. O’Connor via Takashi Koyama.

### Analysis of putative splicing or editing events

RNA was isolated from male flies using TRIzol (Invitrogen) and treated with DNase (Applied extraction kit (Biosystems) according to the manufacturer’s instructions. The isolated RNA was used for cDNA synthesis using the Access RT-PCR system (Promega) with *AMV* reverse transcriptase, and with primers amplifying a 500-bp region surrounding the first stop codon. The PCR products were run on agarose gel electrophoresis and analyzed using Bio-Rad UV-vis camera. Thereafter, the agarose gel band was excised from the gel, DNA extracted using QIAquick gel Qiagen), and used as template for DNA sequencing (Eurofins MWG Operon sequencing service). The following primers were used (5′–3′):

Forward primer: AGGAGGTGGTACGCGTGTGG

Reverse primer: CCTGATTGCCAGCGGAGGAG

### Phylogenetic analysis

Multiple sequence alignments of Dfr from selected species were performed using MAFFT [60]. In cases where SCR was not annotated, the open reading frame immediately downstream of the first stop codon, and in frame, was manually translated into amino acid sequences until the subsequent stop codon to achieve a hypothetical protein extension. The output were used to construct Phylograms in Simple Phylogeny [61] using default parameters including the neighbor-joining method and visualized by real branch lengths. Alignments were additionally imported into MView [62] to obtain the degree of consensus per base.

### Gateway cloning

Different *dfr* expression constructs were made using a 3.7-kb full-length *vvl/dfr* cDNA (provided by W. Johnsson) as template and pENTR^TM^ directional TOPO® cloning kit according to the manufacturer’s instruction (Invitrogen). The following constructs were made: *dfr-3* construct contains 1284 bp cDNA sequence from the start codon to the first TAG stop codon and can solely express Dfr-S; *dfr-4* construct contains the 2142 bp cDNA sequence from the start codon to the second TAG stop codon. It still carries the first stop codon and can express both Dfr-S and Dfr-L, the latter as a result of readthrough. To create an obligate Dfr-L expression construct (*dfr-5*), a point mutation was inserted in *dfr-4*, by inverse PCR with phosphorylated primers, converting the first in-frame TAG stop codon to a lysine codon AAG. The *dfr-6* construct contains the coding sequence between the first and second stop codons (nt 1953 –2810), enabling expression of the 285 amino acid C-terminal extension for antibody production.

The following primers were used (5′–3′):

*dfr-3*, *dfr-4, dfr-5* forward: CACCATGGCCGCGACCTCG

*dfr-6* forward: CACCCAATCAGAAATCCAGG

*dfr-3* reverse: GGCCGCCAACTGATGCGCCG

*dfr-4, dfr-5, dfr-6* reverse: TTCGCCACCCGCTCCGCCCG

The following primers were used to introduce the point mutation (5′–3′):

Forward primer: AAGCAATCAGAAATCCAGGAG

Reverse primer: GTGGGCCGCCAACTGATGCG

Destination plasmids for expression of untagged and tagged constructs of each isoform in cell cultures and bacteria, and for P-element mediated transformation were made via recombination using the Gateway® LR Clonase Enzyme mix according to the manufacturer’s instruction (Invitrogen).

### P-element mediated transformation

P-element-mediated transformation was performed according to Rubin and Spradling [63]. The *pUAS-Dfr-S* and *pUAS-Dfr-L* plasmids were injected together with the Δ2-3 helper plasmid into the recipient strain, *yw* [64]. The eclosed G0 flies were back-crossed with the *yw* flies, and G1 flies were crossed with balancer lines individually to establish stable transformant strains.

### CRISPR /Cas9 gene editing of *dfr/vvl*

The gene editing of *dfr/vvl* was performed using single gRNA according to [65]. Genomic DNA was isolated from the recipient fly strain *vasa::Cas9* line (BL51323). A region of 563 bp around the first in-frame stop codon of the *dfr* gene was amplified by PCR and sequenced to determine potential polymorphism between *vasa::Cas9* line and the reference genome. Microinjections were carried out with 500 ng/μl gRNA plasmid. Injected G0 males were crossed with *w*;; *MKRS/TM6B* balancer stock, 2–3 progeny males from each cross were crossed with *w*;; *MKRS/TM6B* virgins. Stocks were established from the progeny. Homozygous larvae from each stock were chosen for genotyping. Initial experiments and the RNA sequencing was done with homozygous *dfr*^*14*^ that had been outcrossed to *w*;; *MKRS/TM6B*. To further clean up the third chromosome, the *dfr*^*14*^ mutant was crossed with *w*^*1118*^, and F1 females were outcrossed to *w*^*1118*^ background for six generations. Genotyping was performed to trace the mutation in *dfr*.

Oligos for analysis of polymorphism and genotyping (5′–3′) were:

Forward: CAGAAGGAGAAGCGCATGAC

Reverse: TGCTGCTGGTGGTGTTTAAC.

Oligos for gRNA plasmid (5′–3′)

Forward: GCTGCTGCAGCTGAGTTCGACTCC

Reverse: GGAGTCGAACTCAGCTGCAGAAAC

### Immunoprecipitation, in-gel digestion, and mass spectrometry analysis

*Drosophila* S2 cells were transfected with 3 μg of *pAWM-dfr4* using Effectene transfection kit (Qiagen) according to the manufacturer’s instruction. Transfected cells were harvested on day 4 after transfection, washed 2 times in PBS, homogenized in lysis buffer containing 20 mM Tris pH 7.8, 150 mM NaCl, 10 mM MgCl_2_, 2 mM EDTA, 10% Glycerol, 0.5% NP40, 1 mM DTT, and protease inhibitor cocktail according to the manufacturer’s instruction. The homogenate was shaken gently at 4 °C for 10 min and then centrifuged at 1500*g*. Immunoprecipitation was done using mouse anti-Myc antibody (4A6, Millipore) at 1–3 mg/ml and Dynabeads® Protein G (Thermo Fisher Scientific) according to the manufacturer’s instruction. Eluted proteins were separated by 7.5% SDS-polyacrylamide gel electorphoresis. The band corresponding to Dfr-L-Myc was excised manually from a Coomassie-stained gel. In-gel digestion, peptide extraction, MS analysis, and database searches for protein identification were carried out at the Proteomics Biomedicum, Karolinska Institute, Sweden, as follows: In-gel digestion of the gel pieces were done using a MassPREP robotic protein-handling system (Waters, Millford, MA, USA). Gel pieces were destained twice with 100 μl 50 mM ammonium bicarbonate containing 50% acetonitrile at 40 °C for 10 min. The protein was reduced by 10 mM DTT in 100 mM Ambic for 30 min and alkylated with 55 mM iodoacetamide in 100 mM Ambic for 20 min followed by in-gel digestion with 0.3 μg chymotrypsin (modified, Promega, Madison, WI, USA) in 50 mM ammonium bicarbonate for 5 h at 40 °C. Chymotrypsin was used instead of Trypsin due to the relatively sparse number of Arg and Lys in ORF2. The chymotryptic peptides were extracted with 1% formic acid/2% acetonitrile, followed by 50% acetonitrile twice. The liquid was evaporated to dryness and the peptides were injected onto the LC-MS/MS system (Ultimate^TM^ 3000 RSLCnano chromatography system and Q Exactive Plus Orbitrap mass spectrometer, Thermo Scientific). The peptides were separated on a homemade C18 column, 25 cm (Silica Tip 360 μm OD, 75 μm ID, New Objective, Woburn, MA, USA) with a 60 min gradient at a flow rate of 300 nl/min. The gradient went from 5 to 26% of buffer B (2% acetonitrile, 0.1% formic acid) in 55 min and up to 95% of buffer B in 5 min. The effluent was electro-sprayed into the mass spectrometer directly via the column. Peptide mass tolerance was set to ± 10 ppm; fragment mass tolerance: ± 0.02 Da; max missed cleavages : 2. The spectra were analyzed using the Mascot search engine v. 2.4 (Matrix Science Ltd., UK). Protein hits were obtained using SwissProt_202, Decoy data base search. Drosophila (5922 sequences), chymotrypsin, and peptide mass tolerance was set to ± 10 ppm; fragment mass tolerance: ± 0.02; max missed cleavages: 2.

### Antibody production

Antibodies against Dfr-L/ORF2 were raised in rat against a purified recombinant Dfr ORF2 protein (285 amino acids), produced in *E.coli.* Recombinant protein expression, purification, and immunization of rats were carried out by Agrisera AB, Vännäs, Sweden, as follows: GST-tagged Dfr-ORF2 protein was produced in BL21(DE3) and purified by affinity chromatography on a Glutathione Sepharose 4B column. The GST part was cleaved off from the recombinant protein using PreScission Protease (GE Healthcare Life Sciences) according to the manufacturer’s instructions. Purified Dfr-ORF2 protein without the tag was used for immunization of rats. Serum titers were analyzed by immunoassays and antibody specificity against Dfr-L using immunoblot assays.

### Immunocytochemistry of *Drosophila* tissues

*Drosophila* larvae were dissected in phosphate-buffered saline (PBS, pH 7.0) and fixed in 4% paraformaldehyde for 30 min at room temperature. The specimens were washed in PBST (PBS with 0.3% Triton X-100) three times, then blocked in PBST with 0.5% normal goat serum for 1 h at room temperature. Antibody dilutions used were as follows: rat anti-Dfr-S/L (1:400) [29], rat anti-Dfr-L (1:400), guinea pig anti-Neverland (1:1,000) [66], guinea pig anti-Shroud (1:1,000) [67], rabbit anti-Phantom (1:400) [68], rabbit anti-Disembodied (1:400) [68], and rabbit anti-Shadow (1:400) [69]. Secondary antibodies were Alexa Fluor 594 conjugated goat anti-rat (1:500), goat anti-rabbit (1:500), and goat anti-guinea pig (1:500). DAPI was used to stain the nuclei. Flp-out clones were also analyzed using this protocol.

### Immunoblot assays

Protein extraction from dissected tissues was performed as previously described [70]. Extracts were separated by electrophoresis in a 10% SDS-polyacrylamide gel at constant current of 120 volt. Proteins were transferred to polyvinylidinefluoride membranes (Millipore Corporation, Billerica, MA, USA), subsequently blocked 5% dry milk in TBST (Tris Buffered Saline with 0.1% Tween 20) for 1 h at room temperature and then incubated with anti-Dfr S/L, anti-Phm, anti-Dib, or anti-Actin (mAbcam 8224) as primary antibodies, and with ECL^TM^ anti-rat IgG (Amersham), ECL^TM^ anti-mouse IgG (GE Healthcare), and ECL^TM^ anti-rabbit IgG (GE Healthcare) as 2nd antibodies. The blot was developed using either SuperSignal^TM^ West Femto maximum sensitivity substrate or SuperSignal^TM^ West Pico PLUS Chemiluminescent Substrate (Thermo Scientific) according to the manufacturers’ instructions. Digital images were acquired with ChemiDoc™ Imaging Systems (Bio-Rad). Protein levels were quantified with Image Lab™ Software (Bio-Rad) and normalized against Actin or Lamin. Statistics was performed using two-way ANOVA.

### RNA sequence analysis

Total RNA was extracted from BRGCs and bodies of wandering L3 larvae. The body samples were devoid of BRGCs, mouth hooks, and salivary glands. The BRGC and body samples were collected from different larvae respectively and hence considered as separate experiments. Four biological replicates were prepared for each group. The RNA samples were further cleaned up with Qiagen RNeasy kit (Qiagen, Valencia, CA) according to the manufacturer’s instructions. Sequencing was performed at Science for Life Laboratory (National Genomics Infrastructure, Stockholm node), using a HiSeq2500 (Illumina TruSeq Stranded mRNA) with Poly-A selection. Raw data in binary base call (BCL) format were converted to FastQ using bcl2fastq_v2.19.1.403 from the CASAVA software suite. All samples passed the quality test pipeline. High-quality reads per sample were in the range of 19.6–32.1 million, with an average of 25.1 ± 4. Mapped reads per gene (ENSEMBL BDGP6 assembly) were quantified using featureCounts. Datasets from body and BRGC were analyzed separately. Genes with no counts in either group of respective tissue were filtered out from the analysis (7827 in BRGC; 5045 in body) resulting in 9731 (BRGC) and 12,513 (body) remaining. Differences in library sizes between samples where accounted for using the calcNormFactors function to scale reads according to the effective size of each library. Annotation was performed using the Bioconductor 3.8 annotation package org.Dm.eg.db. Differential expression analysis was carried out using Bioconductor 3.8 with the edgeR 3.8 package in R 3.5.2 according to the edgeR user’s guide (26 October 2018 revision; see Additional file 13 for code used). Multidimensional scaling (MDS) of samples was plotted using edgeR using the default setting of leading log-fold-changes between each pair of sample to map the corresponding distances. Venn diagrams were constructed in Photoshop CC 2015. Vulcano plots were constructed using the ggplot2 package in R. Gene ontology (GO) analyses were performed in BRGC or body, respectively, using GOrilla (FDR < 0.1 was considered significant) [71, 72]. As background gene list, all enlisted IDs with expression in at least one of the groups in respective tissue was used. Analyses were performed on upregulated, downregulated, or all differentially expressed hits separately. Redundant GO terms were filtered out using REVIGO [73] with allowed similarity set to “low” (dispensability < 0.5). Generated REVIGO scripts for semantic scatterplots were imported to RStudio for plotting.

### Quantitative RT-PCR

Female virgins of *tub-Gal80*^*ts*^*; phm-Gal4* (200-300 virgins in each bottle) were crossed with *w*^*1118*^*, UAS-dfr-RNAi, UAS-dfr-S*, and *UAS-dfr-L*, respectively. Embryos were collected in a 12-h time window, then maintained at 25 °C. Newly hatched larvae were synchronized and raised at low density (30 larvae/vial) at 18 °C for 4 days, then shifted to 29 °C for 42 h. BRGCs were dissected from the larvae. Ten BRGCs were put into a 1.5-ml tube, flash frozen in liquid nitrogen, then stored at − 80 °C. Three biological replicates were prepared for each genotype. For the quantification of steroidogenic gene expression in *dfr*^*14*^, brain ring gland complexes were dissected from wandering third instar larvae. Four biological replicates were prepared for control *w*^*1118*^ and *dfr*^*14*^. RT-qPCR was performed as previously described [74]. The TaqMan probes are as follows: *phm*, Dm01844265_g1; *nvd*, Dm01844265_g1; *sro*, Dm02146256_g1; *dib*, Dm01843084_g1; *sad*, Dm02139319_g1. The measured transcript levels were normalized relative to *Rpl32* values.

### Flip-out clones

Cell clones were induced as previously described [75] with minor changes. Female virgins *hs-Flp*^*122*^*; UAS-Flp*^*JD1*^*/CyO, Act-GFP*^*JMR1*^*; Act > stop > Gal4, UAS-GFP*^*LL6*^*/TM6b* were crossed with *w*^*1118*^*, UAS-dfr-RNAi, UAS-dfr-S*, and *UAS-dfr-L*, respectively. Embryos were collected in a 24-h time window in vials with normal fly food and extra yeast. A 7–10 min heat shock was applied in a 37 °C water bath 24 h after embryo collection to induce flp-out clones. After clone induction, the vials were placed in a room-temperature water bath for 10 min and then kept at 25 °C. To enable a comparative approach, the specimens of different genotypes incubated with each antibody were analyzed using confocal scanning with identical parameters.

### Ecdysteroid measurements

Ecdysteroid levels were measured with an ELISA kit (20-Hydroxyecdysone Enzyme Immunoassay Kit, ARBOR ASSAYS^TM^) according to the manufacturer’s protocol. Ecdysteroids were extracted followed the protocol in [76]. Briefly, whole animals at the designated time points were homogenized in 0.3 ml methanol by a close fitting pestle, followed by shaking for 4 h, centrifugation at 14,000*g*, and collection of the supernatant. The remaining tissues were re-extracted with 0.3 ml methanol and then with 0.3 ml ethanol. The supernatants were pooled and 0.3 ml was evaporated using SCANVAC (Coolsafe^TM^) freeze dryer followed by re-suspension in Assay Buffer (ARBOR ASSAYS^TM^). Absorbance was measured at 450 nm.

### Cell transfections and luciferase assays

Cell transfections and luciferase assays were performed in *Drosophila* Schneider line-2 cells (S2 cells) as previously described [77] with minor changes. Cells were seeded in 100 μl Schneider’s *Drosophila* medium (GIBCO) in a 96-well plate 1 day before transfection. Cell transfections were performed using the Effectene Transfection Reagent (Qiagen). Two days after transfection, luciferase assays were carried out using the Dual-Luciferase Reporter Assay System (Promega) following the manufacturer’s protocol and analyzed with the EnSpire plate reader (PerkinElmer). The *Actin5C-Gal4* plasmid [77] was used to drive the expression of *UAS-dfr-S*, *UAS-dfr-L, HA-Mld-pUAST* [44] or *UAS-GFP* (as control) together with *pGL3-nvd-Fluc* or *pGL3-spok-Fluc* reporters [44]. The Copia Renilla Control plasmid (#38093; Addgene) [78] was used for measurements of transfection efficiency.

### Statistical analysis

RNA-seq statistical analysis was performed in R using the edgeR package. All other statistics were performed in GraphPad Prism 9. Analysis of Dfr protein band intensities in Act[ts] > Dfr-IR relative to control was performed using a two-way ANOVA with Šidák correction. Distinct Dfr-L::Dfr-S ratios were determined using one-way ANOVA with Tukey’s post hoc test. Differences in larval time to pupariation was determined using Log-rank (Mantel-Cox) test with adjusted significance thresholds according to number of comparisons. Ecdysone titers in *dfr*^*14*^ compared to control was analyzed using multiple *t*-tests, one per time point, with two-stage setup and pooled variance with *Q <* 0.05 considered as significant. For RNA-seq differential expression was detected using the exact test with a false discovery rate (FDR) threshold set to < 0.05 for significant hits. Differential mRNA expression levels between *w[1118]* and *dfr[14]* in the BRGC, obtained from RT-qPCR, were analyzed using multiple unpaired *t*-tests (two-stage step-up, assuming individual variance for each gene), with multiple comparisons based on FDR (*Q <* 0.05 was considered significant). Following *Dfr* misexpression in the BRGC (*phm > GFP/dfr*-IR/*dfr-S*/*dfr-L*), mRNA expression levels were analyzed using two-way ANOVA with Dunnet’s multiple comparison against the control group (*phm > GFP*) post hoc. qRT-PCR data was log2 transformed prior to statistical analysis for homoscedasticity. Relative fluorescence in flp-out clones was analyzed using a one-way ANOVA with Holm-Sidak correction. Data from the luciferase assay were analyzed following log_2_-transformation using one-way ANOVA with Tukey’s correction.

## Supplementary Information

**Additional file 1. **The *dfr/vvl* is a single exon gene with no signs of RNA editing. a No alternative splicing was observed in the *dfr* gene around the first stop codon. Gel electrophoresis of an RT-PCR product of *dfr* mRNA around the first in-frame stop codon. Only a single band was detected. b Sequencing results show no indication of RNA editing around the first stop codon. Upper panel, sequenced with forward primer; lower panel, with reverse primer. Stop codon sequences are boxed.

**Additional file 2.** MS analysis of Dfr-6xMyc following immunoprecipitation. a-b nLC-MS/MS and MASCOT analysis. a Two peaks in the MS spectrum (red boxes) were found to span the Dfr ORF1-ORF2 junction, with the same molecular weight but different charge. b MS/MS fragmentation of precursor m/z 757.36 [M + 3]^+^, with the amino acid sequence AAHXQSEIQESNSAAAASTPASL. Assigned β- and γ-ions are indicated in the figure. c MASCOT analysis against a Drosophila protein reference database (Swiss prot_2020, Drosophila (fruit flies) 5922 sequences) identified m/z 757.36[M + 3]^+^ among 15 other peptides as unique for Dfr (protein score = 154). The identified peptides (of identical sequence), spanning ORF1-ORF2 are highlighted (red box). A second MASCOT analysis was performed using the Dfr-Myc sequence as reference (score significance threshold > 45). Mr (expt), experimental m/z transformed to a relative molecular mass; Mr (calc), relative molecular mass calculated from the matched peptide sequence; ppm, difference (error) between the experimental and calculated masses in parts per million: M, number of missed cleavage sites; Ions score, if there are duplicate matches to the same peptide, then the lower scoring matches are shown in brackets; Expect, expectation value for the peptide match.; R, rank of the peptide match, (1 to 10, where 1 is the best match); U, indicates that the peptide sequence is unique to one protein family member.

**Additional file 3. **Disorder analysis of Dipteran Dfr/Vvl-L proteins. a-d The intrinsic disorder of Dfr/Vvl-L was calculated by the VSL2 algorithm (http://www.pondr.com/). Schematic representation of ORF1 (red), ORF2 (Yellow) and the DNA-binding domains (DBD, blue) are shown above the disorder graph. The horizontal bold line indicates 0.5 disordered score, above which the amino acid sequence is disordered. Dpse, *Drosophila pseudoobscura pseudoobscura*; Gmor, *Glossina morsitans*; Aaeg; *Aedes aegyptii*; Cqui, *Culex quinquefasciatus*; DBD, DNA-binding domain.

**Additional file 4. **Dfr-L is present in several larval and adult tissues. a-h Confocal images of *Drosophila* tissues stained with anti-Dfr-L (red) and DAPI (blue), and a merged image placed above the anti-Dfr-L image of adult brain (a); crop (b); adult salivary gland (c) with an arrow pointing at the tip cells with prominent Dfr-L staining; female oviduct (d) and germarium (e); late stage embryo (f) and boxed region in magnified view (f’), with arrow pointing at the embryonic ring gland; L3 wing imaginal disc (g) and gonad of female white prepupa (h). Scale bars 50 μm. i Summary of anti-Dfr-S/L and anti-Dfr-L staining in control *(w*^*1118*^*)* and *dfr*^*14*^ mutant larval and adult tissues. The fluorescence intensity is represented by -, -/+, +, ++, and +++, from barely detectable to strong.

**Additional file 5.** Processed lists of all genes expressed in either genotype, in body and BRGCs, respectively. F-values, P-values and FDR were derived using the Exact test within the EdgeR package.

**Additional file 6.** Significantly enriched GO-terms from GORILLA and REVIGO online tools.

**Additional file 7.** Expression levels, in Dfr14 relative to control, of genes involved in either ecdysteroid biosynthesis or the response to ecdysone.

**Additional file 8. **Three-dimensional structure of a larval brain-ring gland complex. a Schematic illustration of an L3 larva (upper) and a BRGC, lateral view, anterior to the left. RG, ring gland; BL, brain lobe; VNC, ventral nerve cord; D, dorsal; L, lateral; A, anterior. The ring gland is attached to the brain. It also connects to the spiracles and mouth hooks via the trachea. The bilateral trachea interconnect within the gland. b 3D reconstruction of a larval BRGC, posterior view. *Aug-Gal4 > UAS-GFP* marks the corpus allatum (green), anti-Sad the prothoracic gland (red), and DAPI stains DNA (blue).

**Additional file 9.** Movie of a larval BRGC. The ring gland locates above the brain. The BRGC first rotates 360 degrees along the x axis (horizontal rotation), then rotates 180 degrees along the y axis (vertical rotation). The first image of the movie is in posterior view; the last image is in anterior view (upside down).

**Additional file 10. **Dfr-L overexpression causes defects. a Percentage of pupariation relative to time in hours after larval hatching. *spok-Gal4* was applied to drive Dfr-L overexpression in *dfr*^*14*^ background (*spok-Gal4 > dfr-L; dfr*^*14*^). b Representative images of pupae. Compared to control *w*^*1118*^, the *dfr*^*14*^mutation and Dfr-L overexpression (in control and *dfr*^*14*^ background) increased pupal size. Dfr-L overexpression in *dfr*^*14*^ background caused pupal lethality. c Quantification of relative pupa volume of the genotypes in (b). Bars represent means +SE. Distinct lettering denote significantly different sizes (*p* < 0.05).

**Additional file 11. **Knockdown of *dfr*, as well as overexpression of Dfr-S/L reduces the expression of *nvd, phm, dib, spok* and *sad* mRNA. a. Quantification of mRNA in extracts of BRGCs using RT-qPCR after reducing *dfr* mRNA by RNAi or overexpression of *UAS-dfr-S* or *UAS-dfr-L* in larval PG using the *Phm-Gal4*^*ts*^ driver. Downregulation of *dfr* or *UAS-dfr-S* overexpression significantly reduced the mRNA levels of *nvd, phm, dib, spok* and *sad,* while overexpression of *UAS-dfr-L* had a comparably weaker inhibitory effect on these target genes, albeit significant for all except *nvd* (*p < 0.05, **p < 0.01, ***p < 0.001). b. Upper panel: Immunoblot of protein extracts from S2 cells transfected with a constant amount of *dfr-L* and increasing amounts of *dfr-S* expressing plasmids. Dfr-S protein abundance correlates with increasing concentration of *dfr-S* plasmid, while Dfr-L protein abundance decreased when Dfr-S became predominant, albeit a constant concentration of *dfr-L* expression plasmid was transfected. Actin was used as a loading control. Bottom panel:quantification of relative protein expression levels. c. Schematic illustration of how both downregulation and overexpression of *dfr* may interfere with target gene expression. Upon downregulation (left panel) by RNAi, loss of both Dfr-S and Dfr-L abolishes expression of the ecdysone biosynthesis genes, as in (a). When balanced concentrations of Dfr-S, Dfr-L, Mld and other regulatory factors are present (middle panel) the target genes will be appropriately activated at a high level. The large sphere indicates the formation of an active transcription initiation complex, putatively in the form of a liquid phase condensate. Upon overexpression of either Dfr-S or Dfr-L (right panel), the unbalanced concentrations of regulatory factors will disturb the formation of active transcription initiation complexes, and result in weak target gene activation. In addition, overexpression of Dfr-S causes decreased abundance of Dfr-L, as in (b) and Fig. 8c-d).

**Additional file 12. **Dfr regulates the expression of Nvd, Phm and Sad. a, c, e Prothoracic glands (PGs) carrying GFP-labelled flp-out clones that express different transgenes (control, *UAS-dfr-RNAi*, *UAS-dfr-S*, and *UAS-dfr-L*). Induction of flp-clones as described in Fig. 8. The PGs were stained with anti-Nvd (a), anti-Phm (c) and anti-Sad (e), and shown in magenta (upper panels) or gray (lower panels). Immunofluorescence of Nvd, Phm and Sad was reduced or totally abolished in *UAS-dfr-RNAi*, *UAS-dfr-S*, and *UAS-dfr-L* clones. Scale bars 25 μM. b, d, f Quantification of the relative fluorescence in (a), (c) and (e) respectively (*p < 0.05; **p < 0.01; ***p < 0.001; ****p < 0.0001).

**Additional file 13.** Code used in RNA-seq analysis.

**Additional file 14.** Original immunoblots Fig. 1.

**Additional file 15.** Original immunoblots Fig. 3.

**Additional file 16.** Original immunoblots Fig. 4.

**Additional file 17.** Original immunoblots Fig. 7.

## Data Availability

Sequencing data have been deposited in GEO under the accession number GSE149972 [79]. All other data generated or analyzed during this study are included in the manuscript and additional files, or available from the corresponding author on reasonable request. Material produced as part of this study are available from the corresponding author on reasonable request.
