# Supplementary figures and images for: Stop codon readthrough alters the activity of a POU/Oct transcription factor during Drosophila development
Source: BMC Biol. 2021 Sep 3;19:185. doi: 10.1186/s12915-021-01106-0 (PMC8417969; doi:10.1186/s12915-021-01106-0)

**a**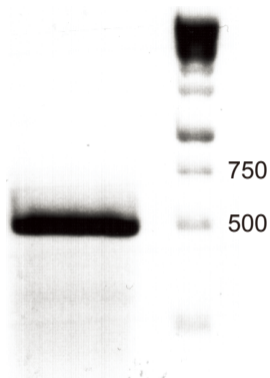**b**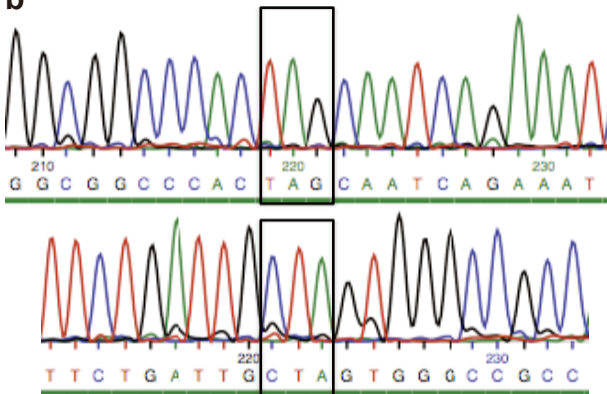

Additional file 1

Supplement: Supplementary file 1 — Additional file 1. The dfr/vvl is a single exon gene with no signs of RNA editing. a No alternative splicing was observed in the dfr gene around the first stop codon. Gel electrophoresis of an RT-PCR product of dfr mRNA around the first in-frame stop codon. Only a single band was detected. b Sequencing results show no indication of RNA editing around the first stop codon. Upper panel, sequenced with forward primer; lower panel, with reverse primer. Stop codon sequences are boxed. [file 12915_2021_1106_MOESM1_ESM.pdf]

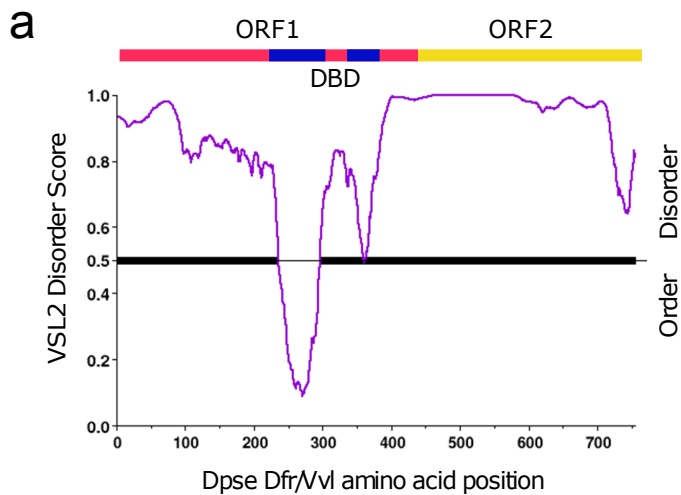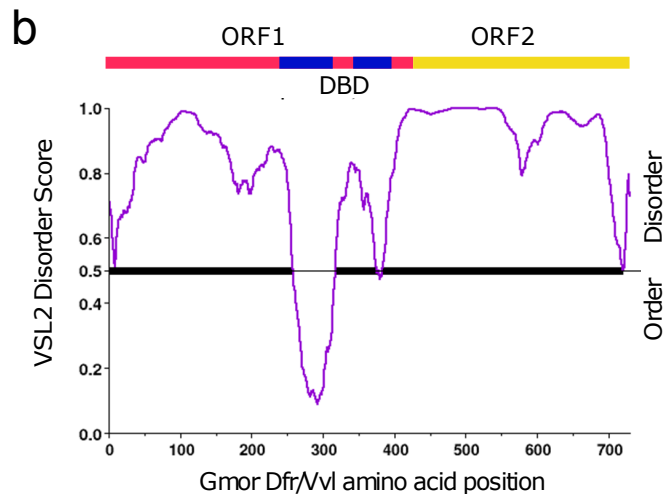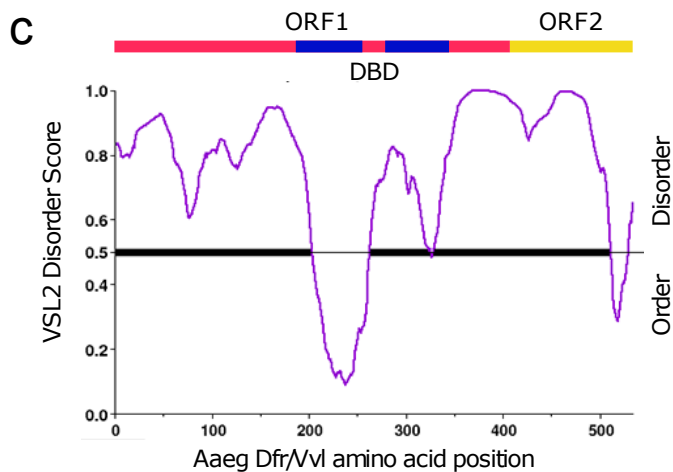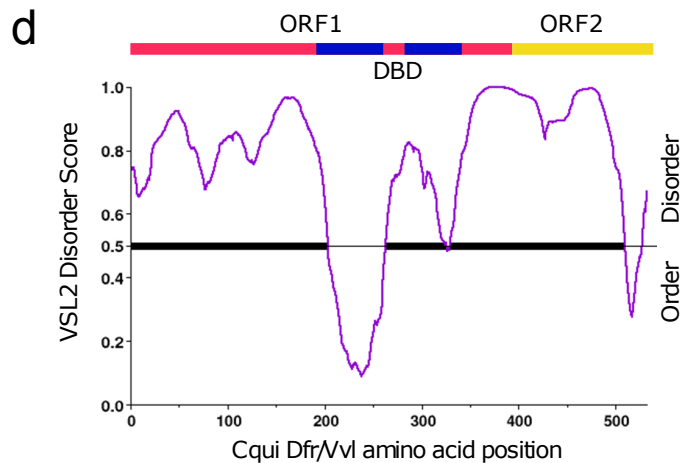

Supplement: Supplementary file 3 — Additional file 3. Disorder analysis of Dipteran Dfr/Vvl-L proteins. a-d The intrinsic disorder of Dfr/Vvl-L was calculated by the VSL2 algorithm (http://www.pondr.com/). Schematic representation of ORF1 (red), ORF2 (Yellow) and the DNA-binding domains (DBD, blue) are shown above the disorder graph. The horizontal bold line indicates 0.5 disordered score, above which the amino acid sequence is disordered. Dpse, Drosophila pseudoobscura pseudoobscura; Gmor, Glossina morsitans; Aaeg; Aedes aegyptii; Cqui, Culex quinquefasciatus; DBD, DNA-binding domain. [file 12915_2021_1106_MOESM3_ESM.pdf]

**a**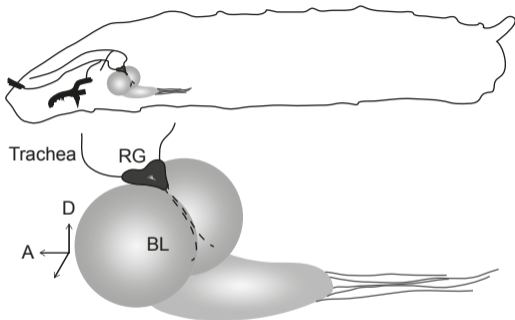**b**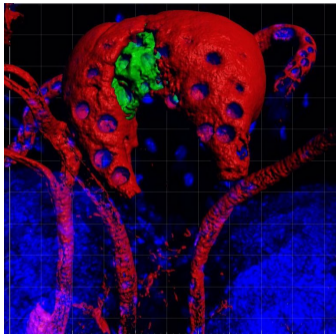

Additional file 8

Supplement: Supplementary file 8 — Additional file 8. Three-dimensional structure of a larval brain-ring gland complex. a Schematic illustration of an L3 larva (upper) and a BRGC, lateral view, anterior to the left. RG, ring gland; BL, brain lobe; VNC, ventral nerve cord; D, dorsal; L, lateral; A, anterior. The ring gland is attached to the brain. It also connects to the spiracles and mouth hooks via the trachea. The bilateral trachea interconnect within the gland. b 3D reconstruction of a larval BRGC, posterior view. Aug-Gal4 > UAS-GFP marks the corpus allatum (green), anti-Sad the prothoracic gland (red), and DAPI stains DNA (blue). [file 12915_2021_1106_MOESM8_ESM.pdf]

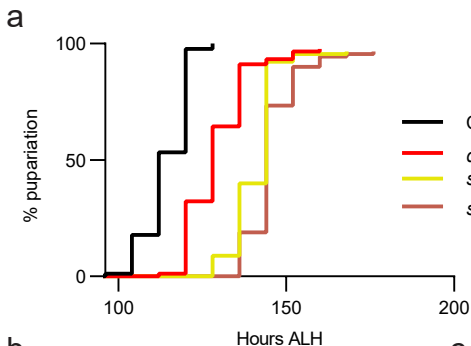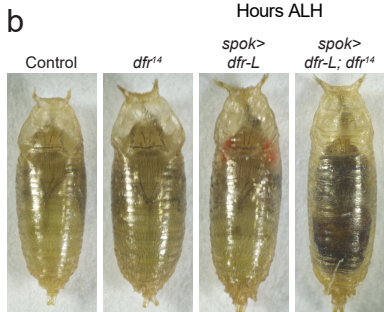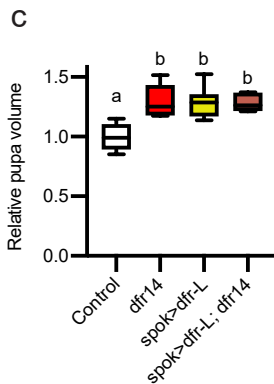

Supplement: Supplementary file 10 — Additional file 10. Dfr-L overexpression causes defects. a Percentage of pupariation relative to time in hours after larval hatching. spok-Gal4 was applied to drive Dfr-L overexpression in dfr14 background (spok-Gal4 > dfr-L; dfr14). b Representative images of pupae. Compared to control w1118, the dfr14mutation and Dfr-L overexpression (in control and dfr14 background) increased pupal size. Dfr-L overexpression in dfr14 background caused pupal lethality. c Quantification of relative pupa volume of the genotypes in (b). Bars represent means +SE. Distinct lettering denote significantly different sizes (p < 0.05). [file 12915_2021_1106_MOESM10_ESM.pdf]

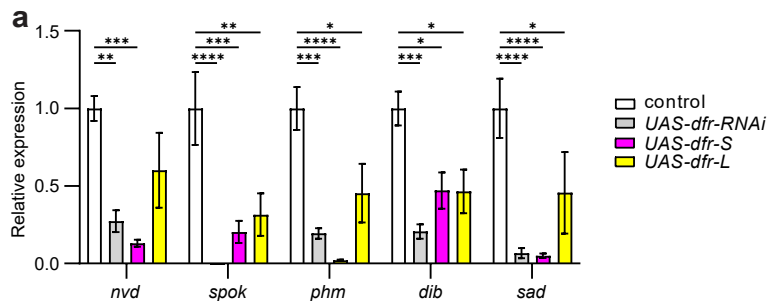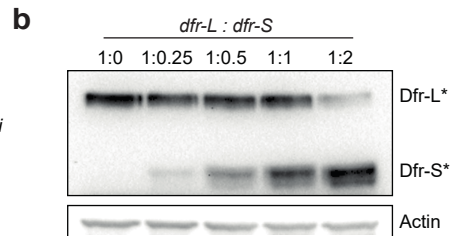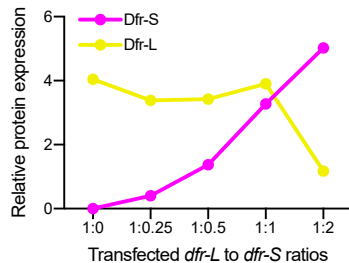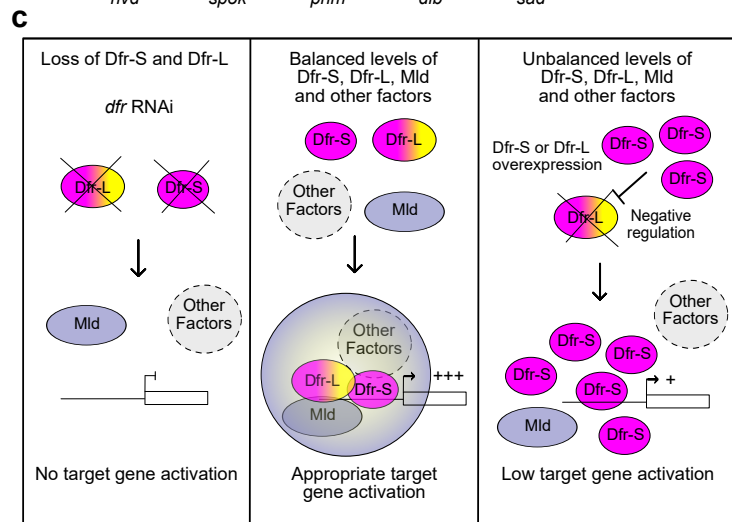

Supplement: Supplementary file 11 — Additional file 11. Knockdown of dfr, as well as overexpression of Dfr-S/L reduces the expression of nvd, phm, dib, spok and sad mRNA. a. Quantification of mRNA in extracts of BRGCs using RT-qPCR after reducing dfr mRNA by RNAi or overexpression of UAS-dfr-S or UAS-dfr-L in larval PG using the Phm-Gal4ts driver. Downregulation of dfr or UAS-dfr-S overexpression significantly reduced the mRNA levels of nvd, phm, dib, spok and sad, while overexpression of UAS-dfr-L had a comparably weaker inhibitory effect on these target genes, albeit significant for all except nvd (*p < 0.05, **p < 0.01, ***p < 0.001). b. Upper panel: Immunoblot of protein extracts from S2 cells transfected with a constant amount of dfr-L and increasing amounts of dfr-S expressing plasmids. Dfr-S protein abundance correlates with increasing concentration of dfr-S plasmid, while Dfr-L protein abundance decreased when Dfr-S became predominant, albeit a constant concentration of dfr-L expression plasmid was transfected. Actin was used as a loading control. Bottom panel:quantification of relative protein expression levels. c. Schematic illustration of how both downregulation and overexpression of dfr may interfere with target gene expression. Upon downregulation (left panel) by RNAi, loss of both Dfr-S and Dfr-L abolishes expression of the ecdysone biosynthesis genes, as in (a). When balanced concentrations of Dfr-S, Dfr-L, Mld and other regulatory factors are present (middle panel) the target genes will be appropriately activated at a high level. The large sphere indicates the formation of an active transcription initiation complex, putatively in the form of a liquid phase condensate. Upon overexpression of either Dfr-S or Dfr-L (right panel), the unbalanced concentrations of regulatory factors will disturb the formation of active transcription initiation complexes, and result in weak target gene activation. In addition, overexpression of Dfr-S causes decreased abundance of Dfr-L, as in (b) and Fig. 8c [file 12915_2021_1106_MOESM11_ESM.pdf]

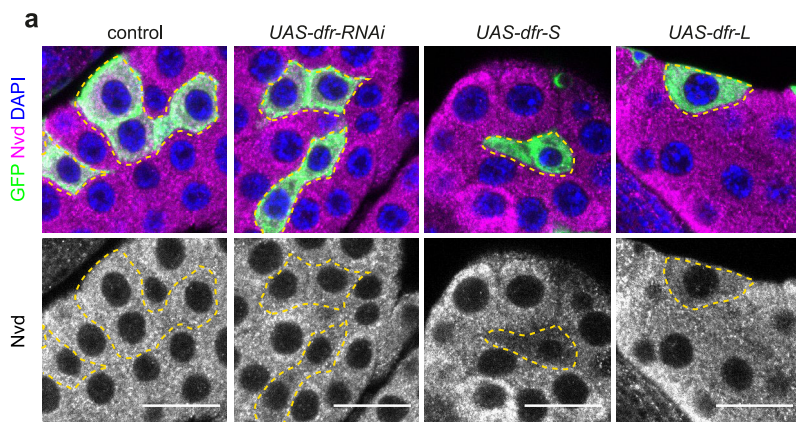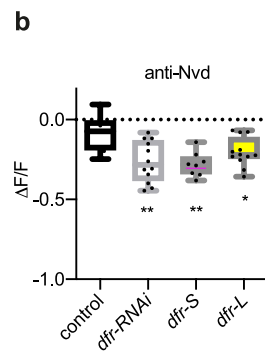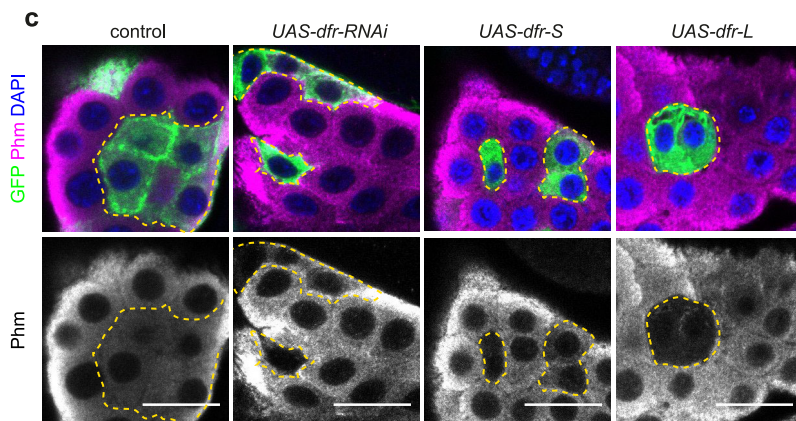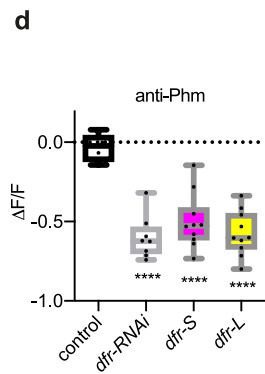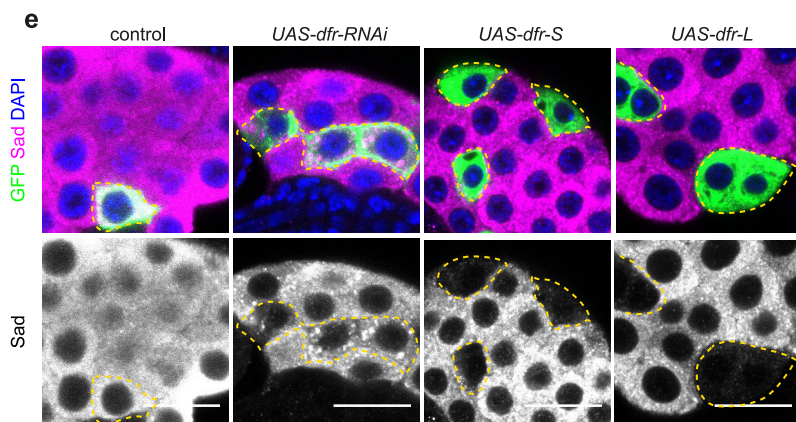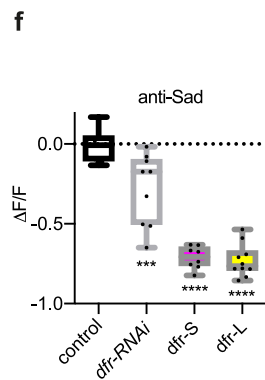

Supplement: Supplementary file 12 — Additional file 12. Dfr regulates the expression of Nvd, Phm and Sad. a, c, e Prothoracic glands (PGs) carrying GFP-labelled flp-out clones that express different transgenes (control, UAS-dfr-RNAi, UAS-dfr-S, and UAS-dfr-L). Induction of flp-clones as described in Fig. 8. The PGs were stained with anti-Nvd (a), anti-Phm (c) and anti-Sad (e), and shown in magenta (upper panels) or gray (lower panels). Immunofluorescence of Nvd, Phm and Sad was reduced or totally abolished in UAS-dfr-RNAi, UAS-dfr-S, and UAS-dfr-L clones. Scale bars 25 μM. b, d, f Quantification of the relative fluorescence in (a), (c) and (e) respectively (*p < 0.05; **p < 0.01; ***p < 0.001; ****p < 0.0001). [file 12915_2021_1106_MOESM12_ESM.pdf]

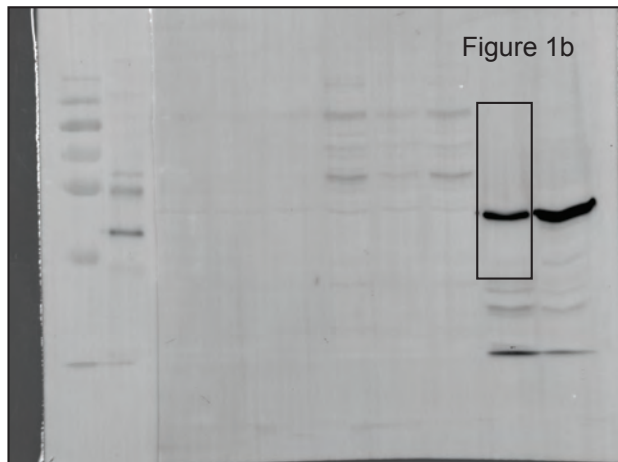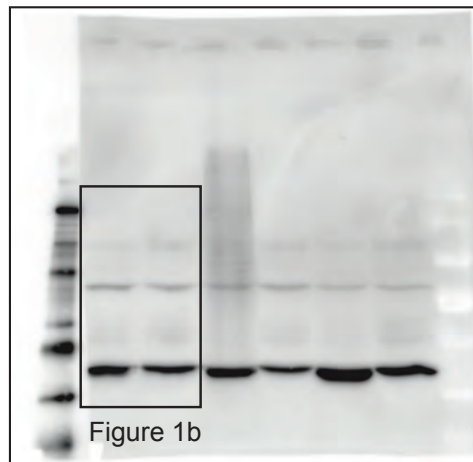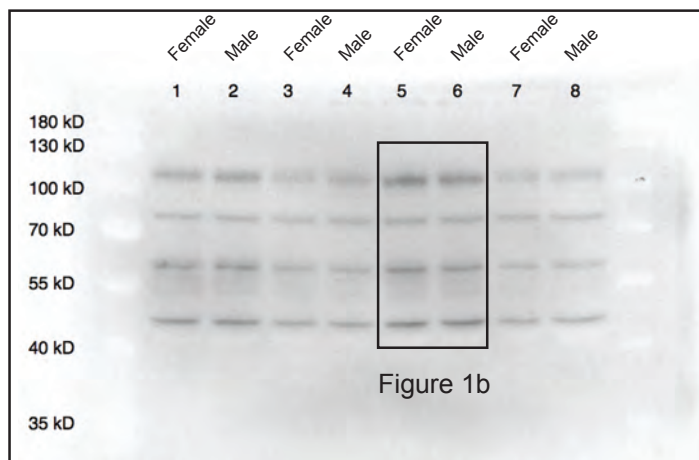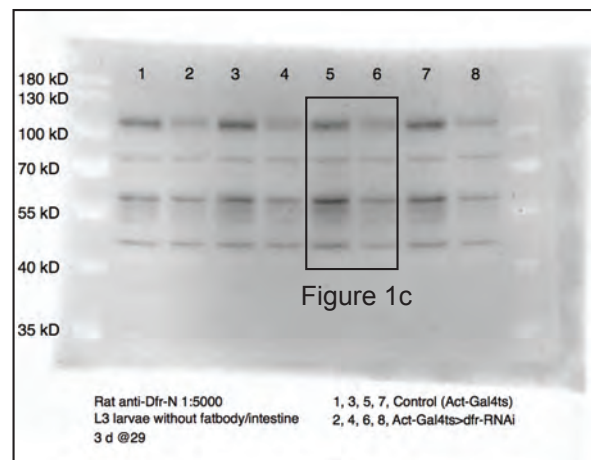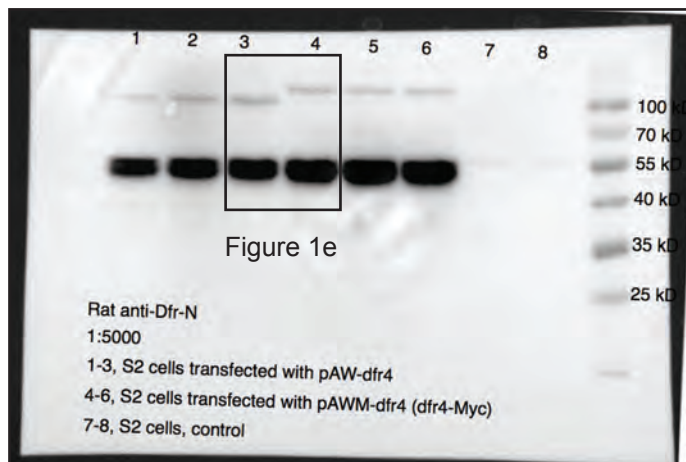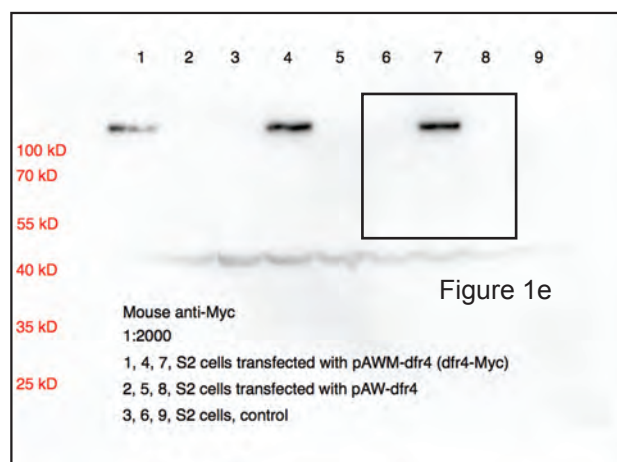

Original Western Blots Fig 1

Supplement: Supplementary file 14 — Additional file 14. Original immunoblots Fig. 1. [file 12915_2021_1106_MOESM14_ESM.pdf]

Figure 3j

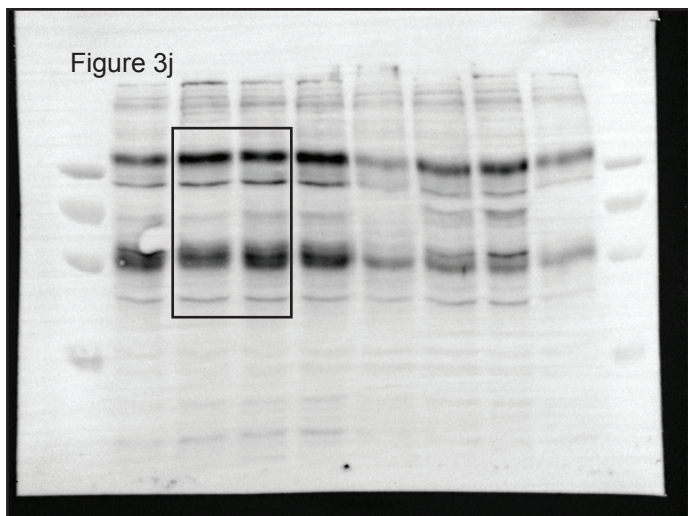

Figure 3k

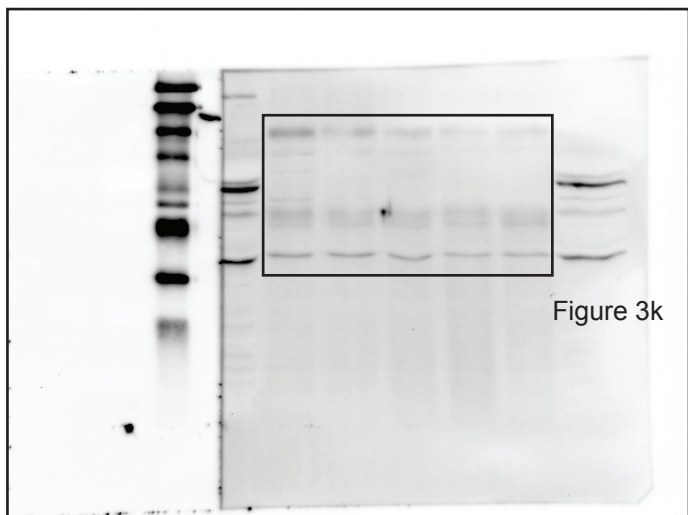

Supplement: Supplementary file 15 — Additional file 15. Original immunoblots Fig. 3. [file 12915_2021_1106_MOESM15_ESM.pdf]

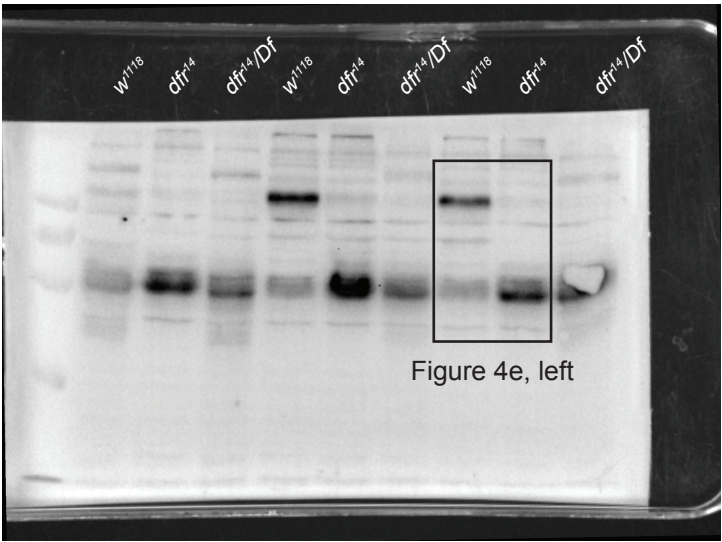

Figure 4e, left

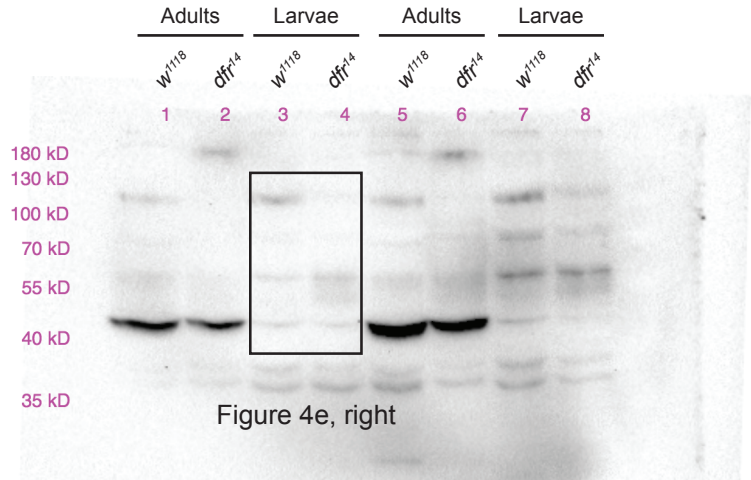

Figure 4e, right

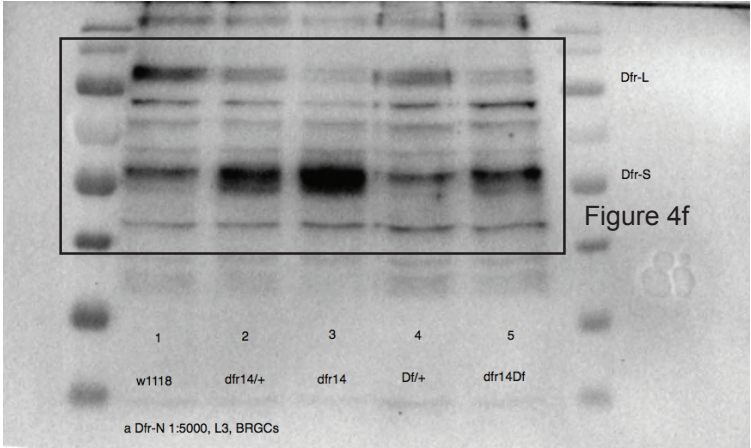

Figure 4f

a Dfr-N 1:5000, L3, BRGCs

Supplement: Supplementary file 16 — Additional file 16. Original immunoblots Fig. 4. [file 12915_2021_1106_MOESM16_ESM.pdf]

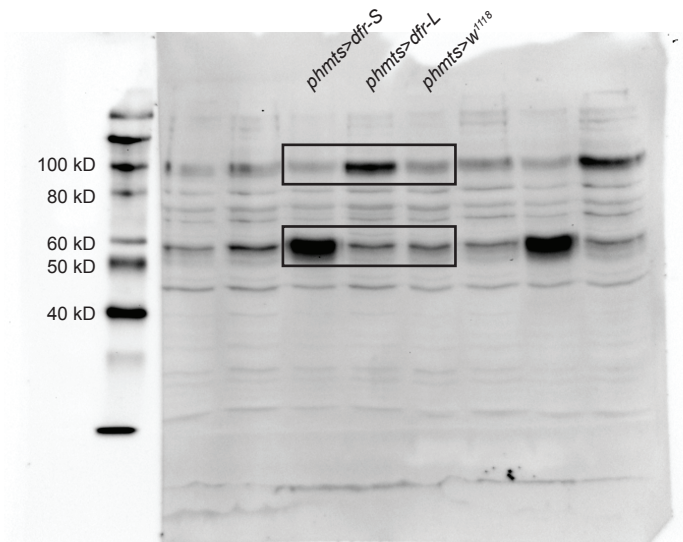

Original Western Blot Fig 7

Supplement: Supplementary file 17 — Additional file 17. Original immunoblots Fig. 7. [file 12915_2021_1106_MOESM17_ESM.pdf]
