# Supplementary material for: Stop codon readthrough alters the activity of a POU/Oct transcription factor during Drosophila development
Source: BMC Biol. 2021 Sep 3;19:185. doi: 10.1186/s12915-021-01106-0 (PMC8417969; doi:10.1186/s12915-021-01106-0)
Supplement: Supplementary file 2 — Additional file 2. MS analysis of Dfr-6xMyc following immunoprecipitation. a-b nLC-MS/MS and MASCOT analysis. a Two peaks in the MS spectrum (red boxes) were found to span the Dfr ORF1-ORF2 junction, with the same molecular weight but different charge. b MS/MS fragmentation of precursor m/z 757.36 [M + 3]+, with the amino acid sequence AAHXQSEIQESNSAAAASTPASL. Assigned β- and γ-ions are indicated in the figure. c MASCOT analysis against a Drosophila protein reference database (Swiss prot_2020, Drosophila (fruit flies) 5922 sequences) identified m/z 757.36[M + 3]+ among 15 other peptides as unique for Dfr (protein score = 154). The identified peptides (of identical sequence), spanning ORF1-ORF2 are highlighted (red box). A second MASCOT analysis was performed using the Dfr-Myc sequence as reference (score significance threshold > 45). Mr (expt), experimental m/z transformed to a relative molecular mass; Mr (calc), relative molecular mass calculated from the matched peptide sequence; ppm, difference (error) between the experimental and calculated masses in parts per million: M, number of missed cleavage sites; Ions score, if there are duplicate matches to the same peptide, then the lower scoring matches are shown in brackets; Expect, expectation value for the peptide match.; R, rank of the peptide match, (1 to 10, where 1 is the best match); U, indicates that the peptide sequence is unique to one protein family member. [file 12915_2021_1106_MOESM2_ESM.pdf]

a

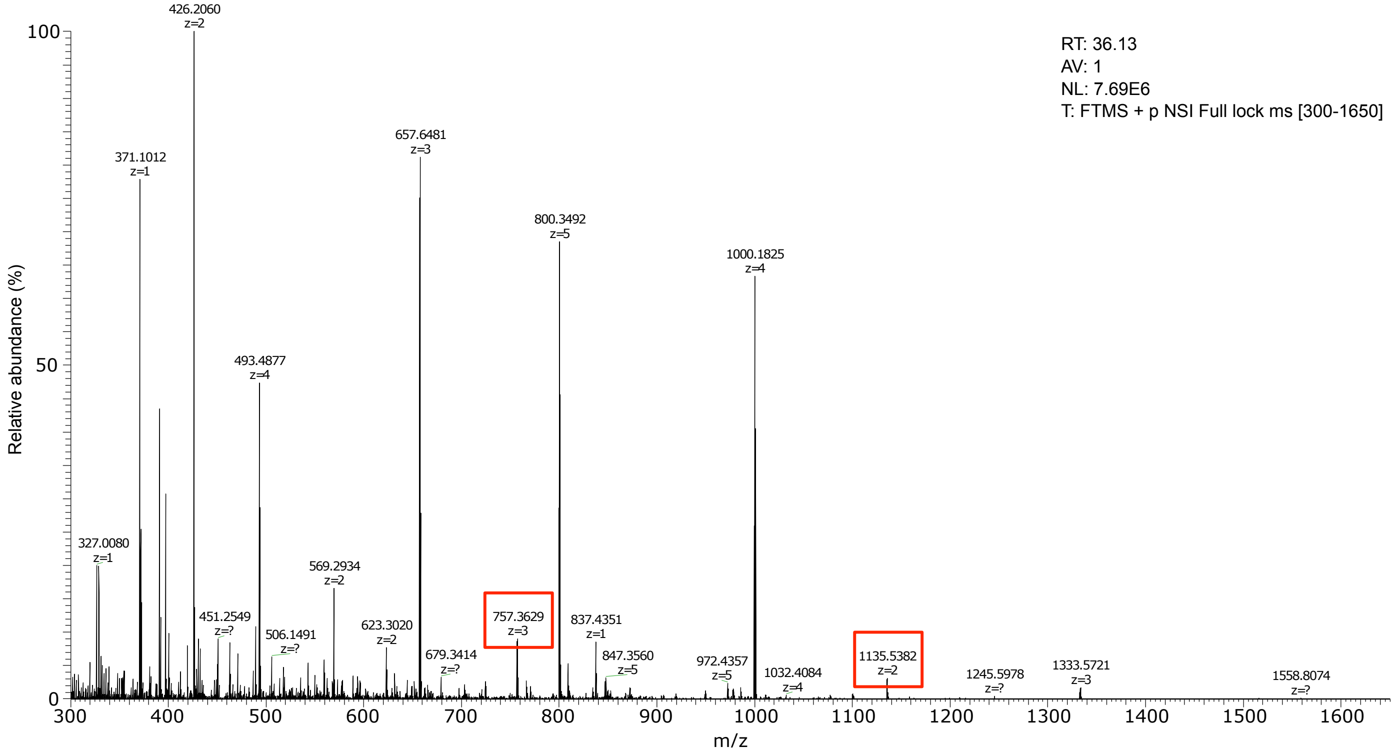

b

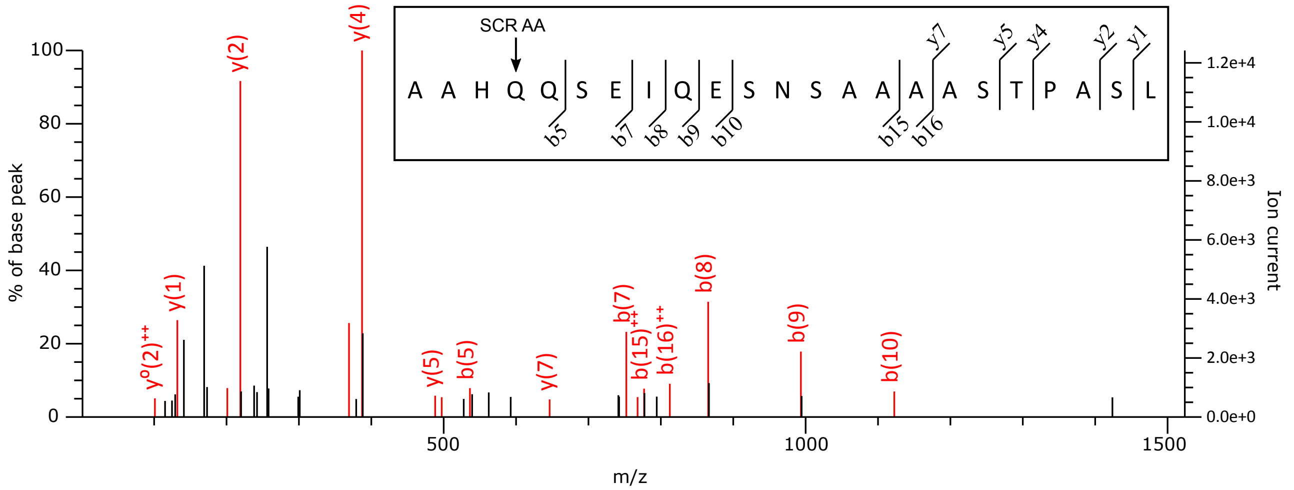

c

Dfr matched peptides	Observed	Mr(expt)	Mr(calc)	ppm	Miss	Score	Expect	Rank	Unique
F.KNMCKL.K	405.2039	808.3932	808.3935	-0.32	0	17	0.022	1	U
Y.MDPMRY.Q	422.6699	843.3252	843.3255	-0.3	0	20	0.01	1	U
W.SALHPDPW.M	461.7245	921.4344	921.4345	-0.02	1	22	0.006	1	U
Y.APTGGSPLQY.H	495.7487	989.4828	989.4818	1.05	1	28	0.0015	1	U
F.SQTTICRF.E	506.748	1011.4814	1011.4808	0.67	0	30	0.00095	1	U
F.GAAAAPGAVRHF.N	562.8019	1123.5892	1123.5887	0.52	0	22	0.0064	1	U
F.GAAAAPGAVRHF.N	375.5372	1123.5898	1123.5887	0.98	0	(14)	0.039	1	U
L.QLEKEVVRVW.F	429.2471	1284.7195	1284.719	0.36	1	22	0.0062	1	U
L.QLEKEVVRVWF.C	478.271	1431.7912	1431.7874	2.61	2	11	0.081	1	U
F.HKQPKPSAQEITSL.A	521.955	1562.8432	1562.8416	0.99	0	18	0.016	1	U
W.HAPHAGHYAPTGGSPL.Q	523.9239	1568.7499	1568.7484	0.92	1	26	0.0027	1	U
Y.MTPPSGDLDMALGGGGY.H	835.8563	1669.698	1669.6964	0.99	2	19	0.012	1	U
L.SQQTRIQQGMASPHAAW.H	638.3087	1911.9043	1911.901	1.73	0	0	0.99	1	U
F.HKQPKPSAQEITSLADSL.Q	650.6819	1949.0239	1949.0218	1.08	1	11	0.074	1	U
L.AAHQQSEIQESNSAAAASTPASL.N	1135.0372	2268.0598	2268.0618	-0.85	0	(27)	0.0022	1	U
L.AAHQQSEIQESNSAAAASTPASL.N	757.0289	2268.0649	2268.0618	1.36	0	45	3E-05	1	U
Y.MDPMRY.Q +2 oxidation (M)	422.6699	843.3252	843.3255	-0.3	0	20	0.01	1	U

Additional file 2
