# Supplementary material for: Stop codon readthrough alters the activity of a POU/Oct transcription factor during Drosophila development
Source: BMC Biol. 2021 Sep 3;19:185. doi: 10.1186/s12915-021-01106-0 (PMC8417969; doi:10.1186/s12915-021-01106-0)
Supplement: Supplementary file 4 — Additional file 4. Dfr-L is present in several larval and adult tissues. a-h Confocal images of Drosophila tissues stained with anti-Dfr-L (red) and DAPI (blue), and a merged image placed above the anti-Dfr-L image of adult brain (a); crop (b); adult salivary gland (c) with an arrow pointing at the tip cells with prominent Dfr-L staining; female oviduct (d) and germarium (e); late stage embryo (f) and boxed region in magnified view (f’), with arrow pointing at the embryonic ring gland; L3 wing imaginal disc (g) and gonad of female white prepupa (h). Scale bars 50 μm. i Summary of anti-Dfr-S/L and anti-Dfr-L staining in control (w1118) and dfr14 mutant larval and adult tissues. The fluorescence intensity is represented by -, -/+, +, ++, and +++, from barely detectable to strong. [file 12915_2021_1106_MOESM4_ESM.pdf]

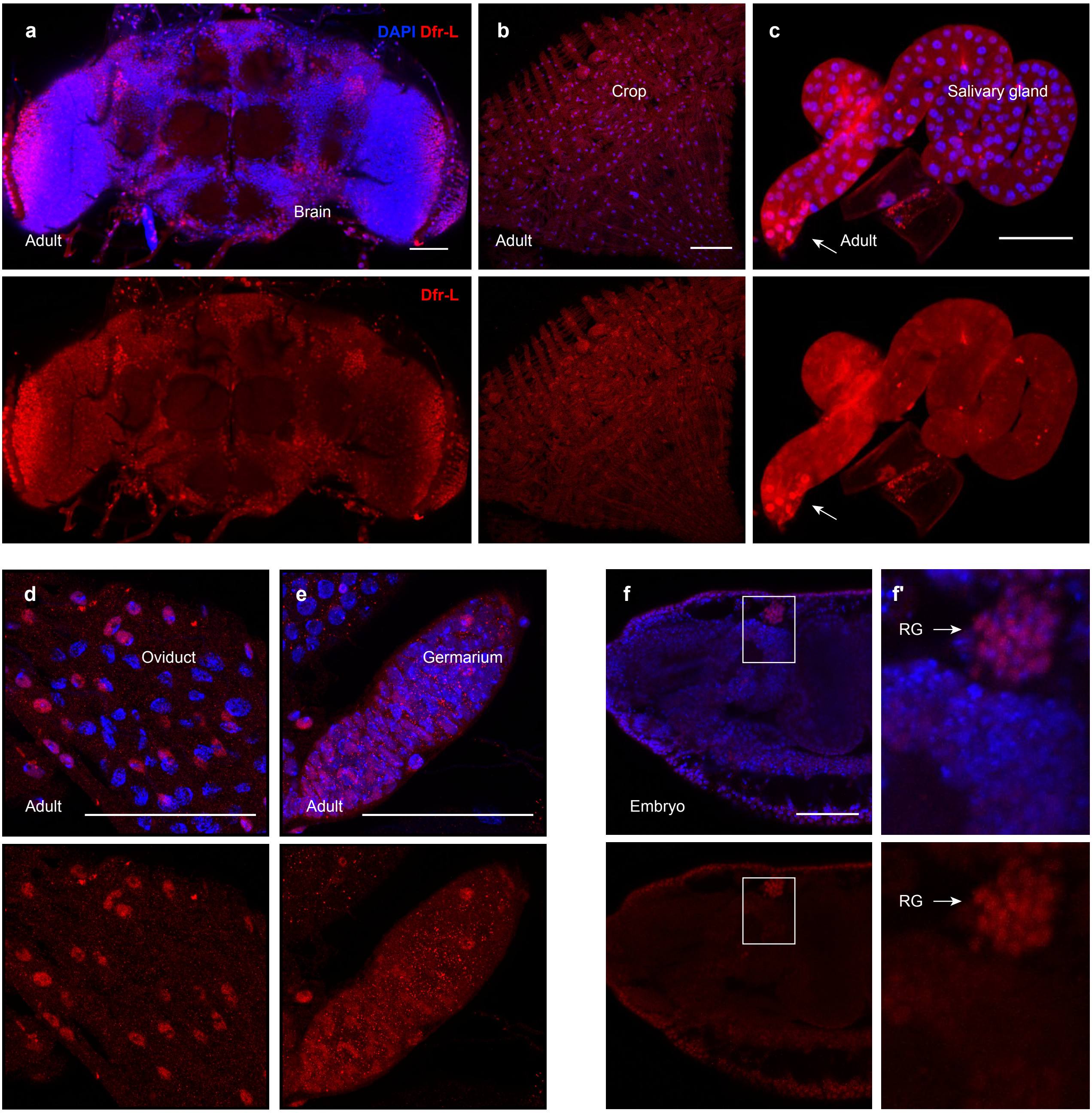

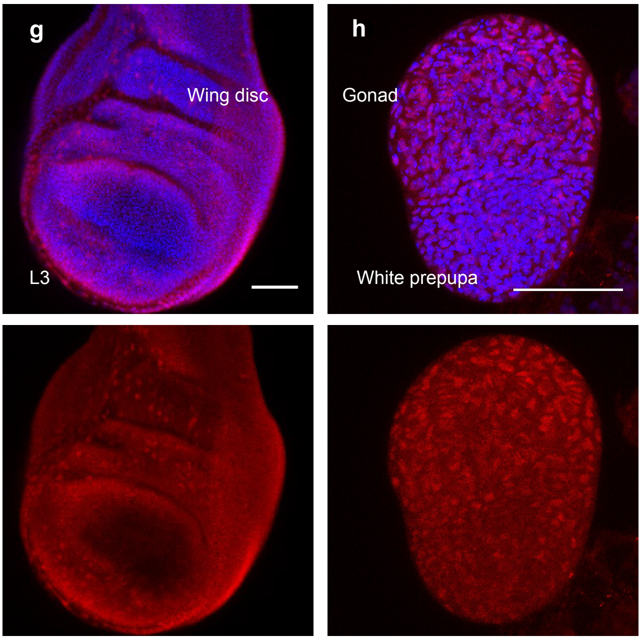

i

	anti-Dfr-S/L		anti-Dfr-L	
	w^{1118}	dfr^{14}	w^{1118}	dfr^{14}
**larva**				
L1 prothoracic gland	+++	+++	+++	-
L2 prothoracic gland	+++	+++	+++	-
L3 prothoracic gland	+++	+++	+++	-
L1 brain	++	++	++	-
L2 brain	++	++	++	-
L3 brain	+	+	+	-
trachea	++	++	++	-
fat body	+	+	+	-
wing disc	+	+	+	-
**adult**				
ejaculatory duct	+++	+++	+	-
testis	+	+	+	-
ovary	+	+	+	-
trachea	++	++	-	-
fat body	++	++	++	-
oenocyte	+++	+++	++	+/-
crop	+	+	+	-
salivary gland	++	++	+	-
ureter	++	++	-	-
brain	++	++	++	+/-
malpighian tubes	+	+	+	-

Additional file 4
