# Supplementary material for: Stop codon readthrough alters the activity of a POU/Oct transcription factor during Drosophila development
Source: BMC Biol. 2021 Sep 3;19:185. doi: 10.1186/s12915-021-01106-0 (PMC8417969; doi:10.1186/s12915-021-01106-0)
Supplement: Supplementary file 13 — Additional file 13. Code used in RNA-seq analysis. [file 12915_2021_1106_MOESM13_ESM.pdf]

```
#EdgeR analysis

# 1 reading input

library("edgeR")

x <- read.delim("bodyraw.txt",row.names="ENSEMBL_ID")

group <- factor(c(1,1,1,1,2,2,2,2))                         #for BRGC, 3 and 4 were concatenated

y <- DGEList(counts=x,group=group)

design <- model.matrix(~group)


#2. Filtering out non-expressed genes

keep <- rowSums(cpm(y)>1) >= 1
y <- y[keep, , keep.lib.sizes=FALSE]
table(keep)

#3. Normalization

y <- calcNormFactors(y)
y$samples

#4. exporting normalized values with annotatations

data.cds <- calcNormFactors(y, method='TMM')
data.norm <- cpm(data.cds, prior.count=2, log=TRUE)

library("AnnotationDbi")

library("org.Dm.eg.db")

res <- as.data.frame(data.norm)

res$symbol <- mapIds(org.Dm.eg.db,

                     keys=row.names(res),

                     column="SYMBOL",

                     keytype="ENSEMBL",

                     multiVals="first")

res$entrez <- mapIds(org.Dm.eg.db,

                     keys=row.names(res),

                     column="ENTREZID",

                     keytype="ENSEMBL",

                     multiVals="first")

res$name =   mapIds(org.Dm.eg.db,

                    keys=row.names(res),

                    column="GENENAME",
```

```
                    keytype="ENSEMBL",

                    multiVals="first")

#5. Estimations of common dispersion and tagwise dispersions

y <- estimateDisp(y)
y$common.dispersion

#6. Exact test

et <- exactTest(y)
topTags(et)


#Vulcano plots, based on gene lists generated from EdgeR analysis and filtered out in Excel

#1. BRGC

library(readxl)

res <- read_excel("brgcvulc.xlsx",

                  col_types = c("text", "numeric", "numeric"))

View(res)

library(calibrate)

with(res, plot(logFC, -log10(FDR), pch=20, font.main=1, main="BRGC", xlim=c(-15.0,15),ylim=c(-
0.0,8), cex.lab=1.5, cex.axis=1.5, cex.main=2, cex.sub=1.5))

with(subset(res, FDR<.05 ), points(logFC, -log10(FDR), pch=20, col="red"))

with(subset(res, FDR<.000000000000000000000000000000000000001 & abs(logFC)>0), textxy(logFC, -
log10(FDR), labs=symbol, cex=.8))

with(subset(res, FDR<.000000000000000000001 & abs(logFC)>5), textxy(logFC, -log10(FDR),
labs=symbol, cex=.8))

#2. Body

res <- read_excel("bodyvulc.xlsx",
                  col_types = c("text", "numeric", "numeric"))
View(res)
library(calibrate)
with(res, plot(logFC, -log10(FDR), pch=20, font.main=1, main="Body", xlim=c(-15.0,15),ylim=c(-0.0
,60), cex.lab=1.5, cex.axis=1.5, cex.main=2, cex.sub=1.5))
with(subset(res, FDR<.05 ), points(logFC, -log10(FDR), pch=20, col="red"))
with(subset(res, FDR<.00000000001 & abs(logFC)>0), textxy(logFC, -log10(FDR), labs=symbol, cex=.8
))
with(subset(res, FDR<.001 & abs(logFC)>8), textxy(logFC, -log10(FDR), labs=symbol, cex=.8))
with(subset(res, FDR<.05 & abs(logFC)>13), textxy(logFC, -log10(FDR), labs=symbol, cex=.8))

#Modifications of the code automatically generated from REVIGO online tool

p1 <- ggplot( data = one.data );
p1 <- p1 + geom_point( aes( plot_X, plot_Y, colour = log10_p_value, size = plot_size), alpha = I(
0.6) ) + scale_size_area();
p1 <- p1 + scale_colour_gradientn( colours = c("blue", "green", "yellow", "red"), limits = c( min
(one.data$log10_p_value), -3), );
```

```
p1 <- p1 + geom_point( aes(plot_X, plot_Y, size = plot_size), shape = 21, fill = "transparent", c
olour = I (alpha ("black", 0.6) )) + scale_size_area();
p1 <- p1 + scale_size( range=c(5, 30)) + theme_bw(); # + scale_fill_gradientn(colours = heat_hcl(
7), limits = c(-300, 0) );
ex <- one.data [ one.data$dispensability < 0.15, ];
p1 <- p1 + labs (y = "semantic space x", x = "semantic space y");
p1 <- p1 + theme(legend.key = element_blank()) ;
one.x_range = max(one.data$plot_X) - min(one.data$plot_X);
one.y_range = max(one.data$plot_Y) - min(one.data$plot_Y);
p1 <- p1 + xlim(min(one.data$plot_X)-one.x_range/10,max(one.data$plot_X)+one.x_range/10);
p1 <- p1 + ylim(min(one.data$plot_Y)-one.y_range/10,max(one.data$plot_Y)+one.y_range/10);
p1 <- p1 + theme(
    axis.line = element_line(size = 1.5),
    axis.text = element_text(size = rel(3)),
    legend.text = element_text(size= rel(1)),
    axis.title = element_text(size = rel(3)))
```
